# Influence of Side Chain–Backbone Interactions and Explicit Hydration on Characteristic Aromatic Raman Fingerprints as Analysed in Tripeptides Gly-Xxx-Gly (Xxx = Phe, Tyr, Trp)

**DOI:** 10.3390/ijms26083911

**Published:** 2025-04-21

**Authors:** Belén Hernández, Yves-Marie Coïc, Sergei G. Kruglik, Santiago Sanchez-Cortes, Mahmoud Ghomi

**Affiliations:** 1 LVTS, INSERM U1148, 74 rue Marcel Cachin, 93017 Bobigny Cédex, France; belen.hernandez@univ-paris13.fr; 2 Institut Pasteur, Université Paris Cité, CNRS UMR 3523, Unité de Chimie des Biomolécules, F-75015 Paris, France; yves-marie.coic@pasteur.fr; 3 Sorbonne Université, CNRS, Inserm, Institut de Biologie Paris-Seine, IBPS, Laboratoire Jean Perrin, LJP, F-75005 Paris, France; sergei.kruglik@upmc.fr; 4 Université Paris Cité, CNRS, Inserm, Laboratoire de Nanomédecine, Biologie Extracellulaire, Intégratome et Innovations en santé, NABI, F-75006 Paris, France; 5 Department of Nuclear, Vibrational and Disordered Media Spectroscopy, Instituto de Estructura de la Materia—Consejo Superior de Investigaciones Cientificas (IEM-CSIC), 28006 Madrid, Spain; s.sanchez.cortes@csic.es

**Keywords:** phenylalanine, tyrosine, tryptophan, tripeptides, glycylphenylalanylglycine, glycyltyrosylglycine, glycyltryptophanylglycine, Raman spectra, density functional theory, implicit and explicit hydration models

## Abstract

Because of the involvement of ***π***-electron cyclic constituents in their side chains, the so-called aromatic residues give rise to a number of strong, narrow, and well-resolved lines spread over the middle wavenumber (1800–600 cm^−1^) region of the Raman spectra of peptides and proteins. The number of characteristic aromatic markers increases with the structural complexity (Phe → Tyr → Trp), herein referred to as (F_i_ = 1, …, 6) in Phe, (Y_i_ = 1, …, 7) in Tyr, and (W_i_ = 1, …, 8) in Trp. Herein, we undertake an overview of these markers through the analysis of a representative data base gathered from the most structurally simple tripeptides, Gly-Xxx-Gly (where Xxx = Phe, Tyr, Trp). In this framework, off-resonance Raman spectra obtained from the aqueous samples of these tripeptides were jointly used with the structural and vibrational data collected from the density functional theory (DFT) calculations using the M062X hybrid functional and 6-311++G(d,p) atomic basis set. The conformation dependence of aromatic Raman markers was explored upon a representative set of 75 conformers, having five different backbone secondary structures (i.e., β-strand, polyproline-II, helix, classic, and inverse γ-turn), and plausible side chain rotamers. The hydration effects were considered upon using both implicit (polarizable solvent continuum) and explicit (minimal number of 5–7 water molecules) models. Raman spectra were calculated through a multiconformational approach based on the thermal (Boltzmann) average of the spectra arising from all calculated conformers. A subsequent discussion highlights the conformational landscape of conformers and the wavenumber dispersion of aromatic Raman markers. In particular, a new interpretation was proposed for the characteristic Raman doublets arising from Tyr (~850–830 cm^−1^) and Trp (~1360–1340 cm^−1^), definitely excluding the previously suggested Fermi-resonance-based assignment of these markers through the consideration of the interactions between the aromatic side chain and its adjacent peptide bonds.

## 1. Introduction

A pioneering work published by Lord and Yu in 1970 [1] emphasized for the first time the importance of aromatic residues in structuring the Raman scattering profiles of peptide chains. In their work, off-resonance Raman spectra of lysozyme compared with those recorded from constituting amino acids showed that aromatic vibrations are responsible for strong, narrow, and well-resolved lines distributed across the middle wavenumber region. Since then, numerous investigations, using both off- and on-resonance Raman spectroscopy, have been devoted to the spectral analysis and environmental sensitivity of aromatic Raman markers [2,3,4,5,6,7,8,9,10,11,12,13], as well as to their use as protein fingerprints in Raman microscopy [14,15].

**Figure 1:**
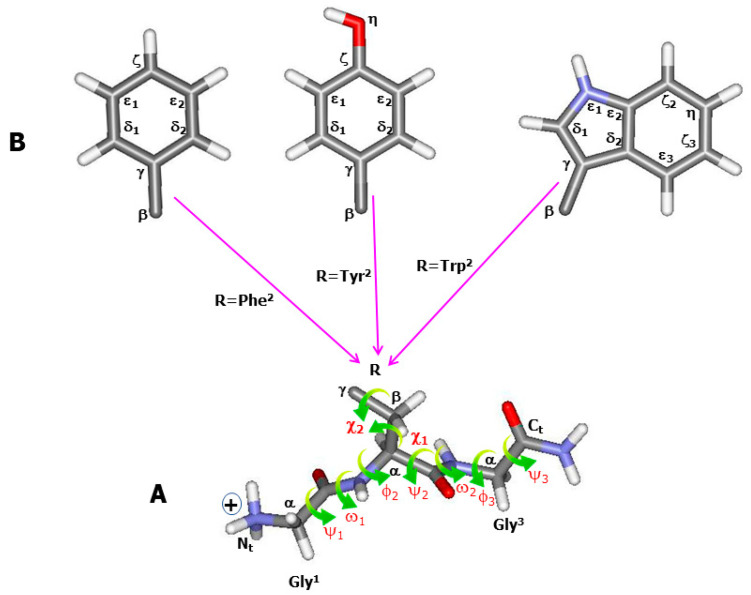
Chemical structure and conformational angles of the cationic species of the three-aromatic-containing tripeptide Gly-Xxx-Gly (Xxx = Phe, Tyr, Trp). (**A**) Definition of the nine torsion angles determining the backbone and side chain conformations. (**B**) The chemical structure and atomic nomenclature of the three aromatic (phenyl, phenol, and indole) rings involved in the chemical structure of Phe, Tyr, and Trp residues. Carbon (grey), nitrogen (blue), oxygen (red), and hydrogen (white). The “+” sign designates the electric charge borne by the terminal NH_3_^+^ group.

This work is limited to the study of the origin and conformational dependence of three aromatic residues, phenylalanine (Phe), tyrosine (Tyr), and tryptophan (Trp) (Figure 1). During recent years, simple molecular compounds, such as free amino acids [16,17,18] and dipeptides [19,20,21], have been used to analyze aromatic vibrational motions. However, it progressively appeared that these short motifs were insufficient to thoroughly reproduce different nonbonded interactions in a peptide chain, especially those between an aromatic side chain and its adjacent peptide bonds. As a result, a tripeptide model with an aromatic residue located at its second position, seemed to be a suitable choice. Herein, we consider the most structurally simple tripeptides with the primary sequence Gly-Xxx-Gly (Xxx = Phe, Tyr, Trp) (Figure 1), because the two flanking Gly residues at either side of the aromatic residue do not provide characteristic vibrations overlapping with the aromatic Raman markers [22,23]. On the other hand, the structuring trend of tripeptides in aqueous solution has received attention during the two past decades. Among these studies, a phenomenological approach applied to homo- and hetero-tripeptides revealed the capability of these compounds to form ordered secondary structures, such as β-like, helical, polyproline-II (pP-II), and γ-turn folds [24,25,26,27,28,29,30]. To explore as accurately as possible the structural and vibrational features of short peptides’ quantum mechanical approaches, and more commonly those based on the density functional theory (DFT), [31] has been applied. However, the main objective in DFT calculations is to choose suitable functionals [32]. Although semiempirical (parameterized) functionals have shown a higher flexibility to account for the experimental data, the shortcomings of the widely used B3LYP functional [33,34], albeit a three-parameter functional, has been revealed in relation to the treatment of noncovalent interactions in molecular systems. A systematic investigation based on the use of a series of hybrid functionals [23,35] has recently shown that improved hybrid functionals, such as ωB97XD [36,37,38] and M062X [39], provide better structural stability in tripeptides. However, it has also been evidenced [23] that the relative energies obtained by these functionals are systematically higher than those predicted by the Møller–Plesset (MP2) method [40] taken as a reference. To account for the solvent effects on the structural features, several hydration models were considered, either by embedding the Gly-rich tripeptides [23,41,42,43] in a polarizable solvent continuum (PCM) [44,45], or by surrounding the cationic Ala-rich [35,46] and Gly-rich [35,43,47] tripeptides with a number of explicit water molecules.

Our recent DFT calculations on both Gly-rich and Ala-rich tripeptides [35,43] have shown that the structural stability of all types of conformers, especially those with an inverse γ-turn conformation, is preserved upon geometry optimization by means of the M062X functional, provided that (i) they are explicitly hydrated by a minimal number of water molecules interacting preferentially with the polar sites of backbone and aromatic side chains and (ii) the whole cluster formed by each tripeptide conformer and its hydrating molecules is embedded in a solvent continuum. Our objective in the present work was to extend this protocol to the three tripeptides Gly-Xxx-Gly (Xxx = Phe, Tyr, Trp) upon consideration of different types of secondary structures (β-like, helix, pP-II, and γ-turn) and plausible side chain orientations. The structural and vibrational data of the optimized conformers, as well as the corresponding relative energies, were subsequently used to calculate the thermal averaged Raman spectra through multiconformational analysis. The reliability of the theoretical spectra was checked through their comparison with the experimental ones obtained from the solution samples. It was shown that beyond the validation of the used theoretical methods, the confrontation of the experimental and calculated Raman spectra leads to obtaining insight into the energetic and conformational landscape of each tripeptide in an aqueous environment.

## 2. Results

### 2.1. Observed Solution Raman Spectra

**Figure 2:**
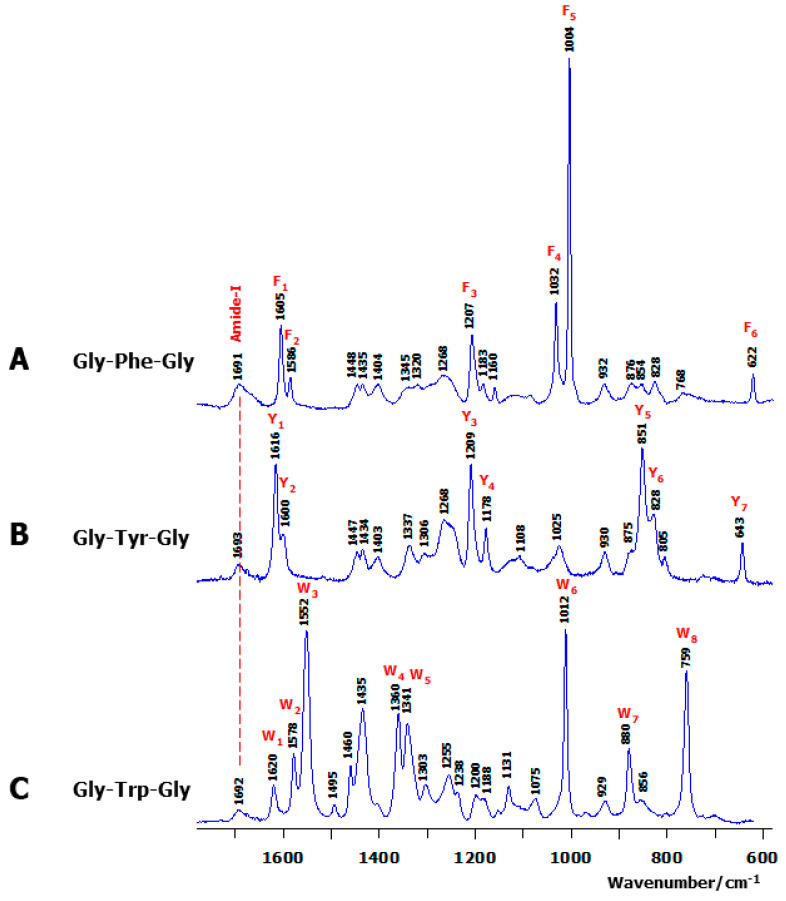
Room-temperature Stokes Raman spectra of the cationic species of the tripeptide Gly-Xxx-Gly (Xxx = Phe, Tyr, Trp) observed in aqueous samples. Each spectrum is buffer and counterion (TFA) subtracted. (**A**) Raman spectrum of Gly-Phe-Gly, on which the positions of the six characteristic Phe Raman markers (F_i_, i = 1, …, 6) are marked in red colour. (**B**) Raman spectrum of Gly-Tyr-Gly, on which the positions of the Tyr Raman markers (Y_i_, i = 1, …, 7) are marked in red colour. (**C**) Raman spectrum of Gly-Trp-Gly, on which the positions of the eight characteristic Trp Raman markers (W_i_, i = 1, …, 8) are marked in red colour.

Buffer- and TFA-subtracted Raman spectra recorded from solution samples of the three tripeptides are displayed in Figure 2 within the middle wavenumber spectral region. Because of the shortness of the backbone, including only three amide bands (two inter-residue and another located at the C^ter^ terminus) (Figure 1A), only a weak, broad amide-I band centred at ~1692 cm^−1^ was observed in all tripeptides (Figure 2). The number of aromatic markers, giving rise to intense and narrow Raman lines, increases with the aromatic ring structural complexity (phenyl → phenol → indole), referred to as (F_i_ = 1, …, 6) in Gly-Phe-Gly (Figure 2A), (Y_i_ = 1, …, 7) in Gly-Tyr-Gly (Figure 2B), and (W_i_ = 1, …, 8) in Gly-Trp-Gly (Figure 2C). See also Table 1 for the corresponding wavenumbers. Isotopic shifts in aromatic markers upon H-D exchange on labile hydrogen atoms were previously reported [41,42,43].

### 2.2. Theoretical Conformers and Raman Spectra

Tables S1–S3 (Supporting Information) provide the energetic and structural data, i.e., relative energy (ΔE) and backbone and side chain torsion angles of optimized conformers, as well as intramolecular H-bond lengths (d_HB_) corresponding to folded structures. The energy interval, within which the ΔE values are located, increases with the structural complexity of the aromatic ring: 0 ≤ ΔE ≤ 7.66 kcal/mol in Gly-Phe-Gly (Table S1); 0 ≤ ΔE ≤ 8.43 kcal/mol in Gly-Tyr-Gly (Table S2); and 0 ≤ ΔE ≤ 10.72 kcal/mol in Gly-Trp-Gly (Table S3). All conformers, initially having a β-strand structure, were transformed into extended chains, i.e., with a quite flat and quasi-planar backbone. Helical, pP-II, and γ-turn structures are maintained upon geometry optimization, as confirmed by the (Φ_2_,ψ_2_) torsion angles corresponding to the middle (aromatic) residue.

**Figure 3:**
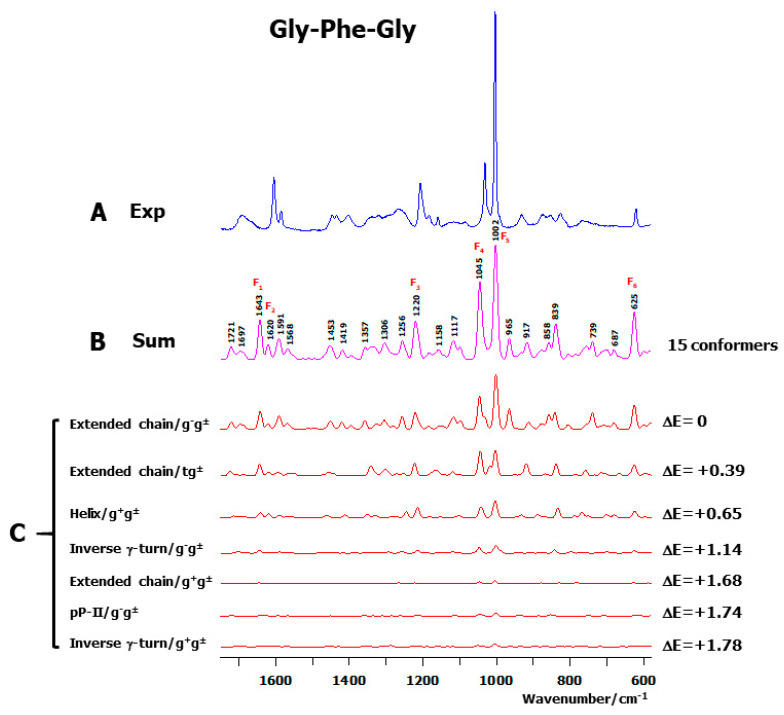
Comparison between the experimental and calculated Raman spectra of the cationic species of the tripeptide Gly-Phe-Gly in the middle wavenumber spectral region. (**A**) Room-temperature solution Raman spectrum (Exp). For the positions of the Raman bands, see Figure 2A. (**B**) Thermal-averaged Raman spectrum (Sum) obtained from the 15 conformers of the tripeptide, interacting with five water molecules and embedded in a solvent continuum. (**C**) Thermal-weighted calculated spectra corresponding to the low-energy conformers with ΔE ≤ 2 kcal/mol. The backbone and side chain conformations of each conformer are reported at the left side, and the relative energy (ΔE in kcal/mol) is displayed at the right side of each spectrum.

**Figure 4:**
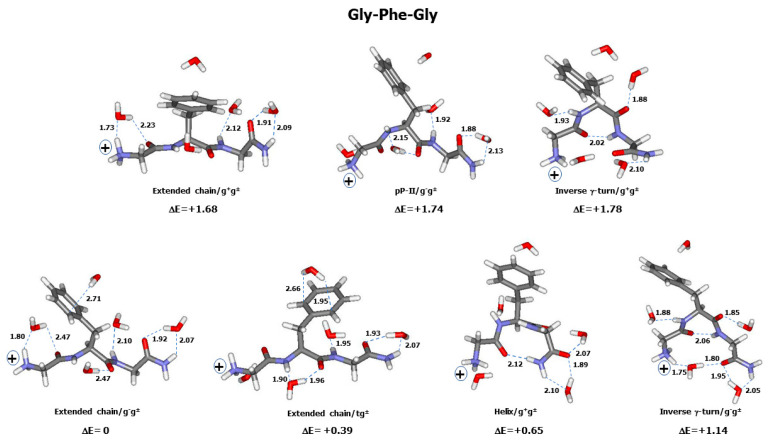
Graphic representation of the conformers of the tripeptide Gly-Phe-Gly with ΔE ≤ 2 kcal/mol. Each conformer is surrounded by five explicit water molecules and embedded in a solvent continuum. Backbone and side chain conformations, as well as relative energy (ΔE in kcal/mol), are mentioned below each conformer. See Table S1 (Supporting Information) for conformational angles. Carbon (grey), nitrogen (blue), oxygen (red), and hydrogen (white). The “+” sign designates the electric charge borne by the terminal NH_3_^+^ group. Intra- and inter-molecular H-bonds are drawn with broken green lines, for which the lengths (in Å) are reported.

The experimental and calculated Raman spectra of Gly-Phe-Gly are compared in Figure 3A,B. The calculated spectrum is the thermal (Boltzmann) average of the individual spectra obtained from 15 optimized conformers (Table S1, Supporting Information). To better emphasize the main contributing conformers, the thermal-weighted calculated spectra corresponding to the low-energy conformers with a ΔE ≤ 2 kcal/mol are displayed in Figure 3C. This proves that the contribution of the higher-energy conformers (with ΔE > 2 kcal/mol) to the global Raman intensity is negligible. The graphical representation of the seven low-energy conformers (ΔE < 2 kcal/mol) is displayed in Figure 4.

**Figure 5:**
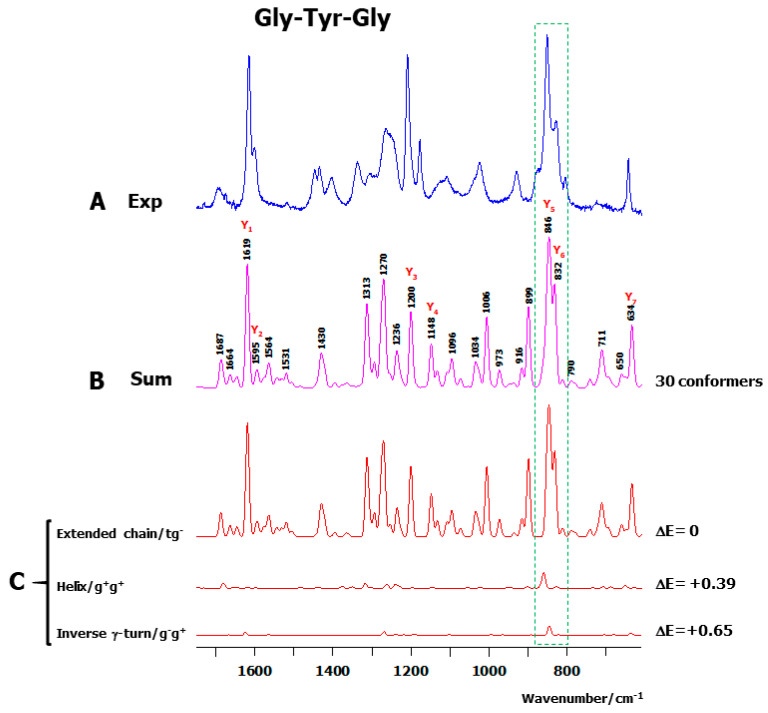
Comparison between the experimental and calculated Raman spectra of the cationic species of the tripeptide Gly-Tyr-Gly in the middle wavenumber spectral region. (**A**) Room-temperature solution Raman spectrum (Exp). For the positions of the Raman bands, see Figure 2B. (**B**) Thermal-averaged Raman spectrum (Sum) obtained from the 30 conformers of the tripeptide, interacting with six water molecules and embedded in a solvent continuum. (**C**) Thermal-weighted calculated spectra corresponding to the low-energy conformers with ΔE ≤ 2 kcal/mol. The backbone and side chain conformations of each conformer are reported at the left side, and the relative energy (ΔE in kcal/mol) is displayed at the right side of each spectrum.

**Figure 6:**
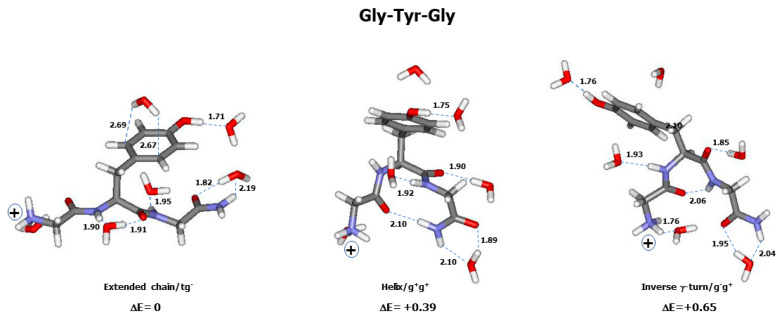
Graphic representation of the conformers of the tripeptide Gly-Tyr-Gly having ΔE ≤ 2 kcal/mol. Each conformer is surrounded by 6 explicit water molecules and embedded in a solvent continuum. Backbone and side chain conformations, as well as relative energy (ΔE in kcal/mol) are mentioned below each conformer. See Table S2 (Supporting Information) for conformational angles. Carbon (grey), nitrogen (blue), oxygen (red), hydrogen (white). “+” sign designates the electric charge borne by the terminal NH_3_^+^ group. Intra- and inter-molecular H-bonds are drawn with broken green lines, of which the lengths (in Å) are reported.

Similarly, the comparison between the observed and calculated (thermal average of 30 conformers) Raman spectra of Gly-Tyr-Gly is shown in Figure 5A,B. Only three conformers were found within the ΔE ≤ 2 kcal/mol range. The thermal corrected Raman spectra of these three conformers are shown in Figure 5C (see graphical representation of these conformers in Figure 6).

**Figure 7:**
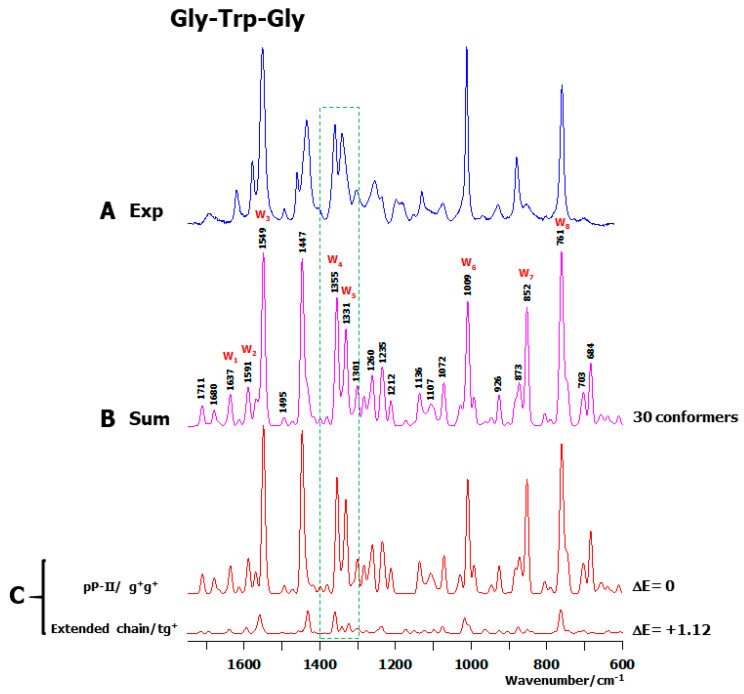
Comparison between the experimental and calculated Raman spectra of the cationic species of the tripeptide Gly-Trp-Gly in the middle wavenumber spectral region. (**A**) Room-temperature solution Raman spectrum (Exp). For the positions of the Raman bands, see Figure 2C. (**B**) Thermal-averaged Raman spectrum (Sum) obtained from the 30 conformers of the tripeptide, interacting with seven water molecules and embedded in a solvent continuum. (**C**) Thermal-weighted calculated spectra corresponding to the low-energy conformers with ΔE ≤ 2 kcal/mol. The backbone and side chain conformations of each conformer are reported at the left side, and the relative energy (ΔE in kcal/mol) is displayed at the right side of each spectrum.

**Figure 8:**
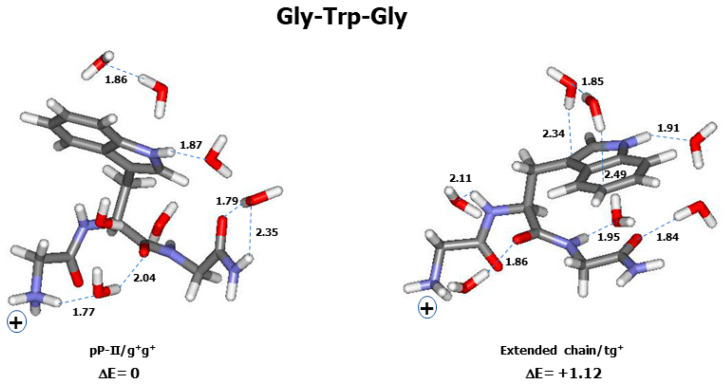
Graphic representation of the conformers of the tripeptide Gly-Trp-Gly with ΔE ≤ 2 kcal/mol. Each conformer is surrounded by seven explicit water molecules and embedded in a solvent continuum. Backbone and side chain conformations, as well as relative energy (ΔE in kcal/mol) are mentioned below each conformer. See Table S3 (Supporting Information) for conformational angles. Carbon (grey), nitrogen (blue), oxygen (red), and hydrogen (white). The “+” sign designates the electric charge borne by the terminal NH_3_^+^ group. Intra- and inter-molecular H-bonds are drawn with broken green lines, for which the lengths (in Å) are reported.

At last, the comparison between the observed and calculated (thermal average of 30 optimized conformers) Raman spectra of Gly-Trp-Gly is shown in Figure 7A,B. Only two conformers are located within the ΔE ≤ 2 kcal/mol range (Figure 7C). See Figure 8 for the graphical representation of these conformers.

## 3. Discussion

### 3.1. Conformational and Energetic Landscapes of Tripeptides

The relative energy (ΔE) of a conformer was shown to depend on its backbone structure, as well as on the aromatic side chain type (phenyl, phenol, indole) and orientation (Tables S1–S3, Supporting Information). For instance, in Gly-Phe-Gly, the lowest-energy conformer (ΔE = 0) is an extended chain with a g^−^g^±^ side chain orientation (Figure 4), whereas in Gly-Tyr-Gly, an extended chain with a tg^−^ side chain corresponds to the lowest energy. In Gly-Trp-Gly, the lowest-energy conformer was found to have a pP-II backbone with a g^+^g^+^ side chain (Figure 8). However, in all tripeptides, classic γ-turn conformers provide the highest ΔE values, whereas helical and inverse γ-turn structures form the intermediate relative energies.

In a helical structure, an intramolecular H-bond is formed between the backbone C=O (Gly^1^) and one of the hydrogen atoms involved in the C^ter^ amide group (Figure 2 and Figure 4). Its length (d_HB_) increases with the aromatic ring’s structural complexity, i.e., 2.09–2.12 Å in Gly-Phe-Gly, 2.07–2.24 Å in Gly-Tyr-Gly, and 2.02–2.27 Å in Gly-Trp-Gly. In γ-turn conformers, the intramolecular H-bond, also referred to as the “turn closing H-bond”, is formed between the backbone C=O (Gly^1^) and N-H (Gly^3^) (Figure 2 and Figure 4). Surprisingly, the d_HB_ values relative to classic γ-turn folds are generally shorter than those corresponding to inverse γ-turn folds: 1.91–2.01 Å (classic γ-turn) versus 2.02–2.27 Å (inverse γ-turn) in Gly-Phe-Gly; 1.90–2.07 Å (classic γ-turn) versus 2.00–2.27 Å (inverse γ-turn) in Gly-Tyr-Gly; and 1.78–2.00 Å (classic γ-turn) versus 2.07–2.37 Å (inverse γ-turn) in Gly-Trp-Gly. As already mentioned, conformers with a classic γ-turn fold have systematically higher ΔE values than those assigned to inverse γ-turn structures. This means that the shortness of intramolecular H-bonds cannot solely explain the higher stability of conformers, and favourable (versus unfavourable) nonbonded interactions occurring between the aromatic ring and the backbone of a tripeptide play the key role in forming the energy landscape of its conformers.

The influence of an aromatic ring’s orientation on relative energies can be better described by considering the role of the side chain torsion angles (χ_1_, χ_2_) (Figure 1A). χ_1_ orientation places the C_γ_ atom of an aromatic ring with respect to the backbone. As a result, with a χ_1_(g^−^) orientation, the aromatic ring is placed onto the peptide bond located at the N^ter^ side of the aromatic residue, whereas with a χ_1_(t), the opposite orientation is favoured, enabling aromatic ring interactions with the peptide bond at its C^ter^ side. At last, a χ_1_(g^+^) orientation brings the aromatic ring between the two aforementioned peptide bonds (Figure 4). The role of χ_2_ is simply maintaining the aromatic ring stacked with the backbone. Consequently, the side chain orientation defined by the pair (χ_1_, χ_2_) leads to the optimization of the interactions between the aromatic ring and its adjacent peptide bonds. On the other hand, the orientation change in the aromatic ring brings drastic modification to the location of the water molecules, thus affecting the relative energies of hydrated conformers.

**Figure 9:**
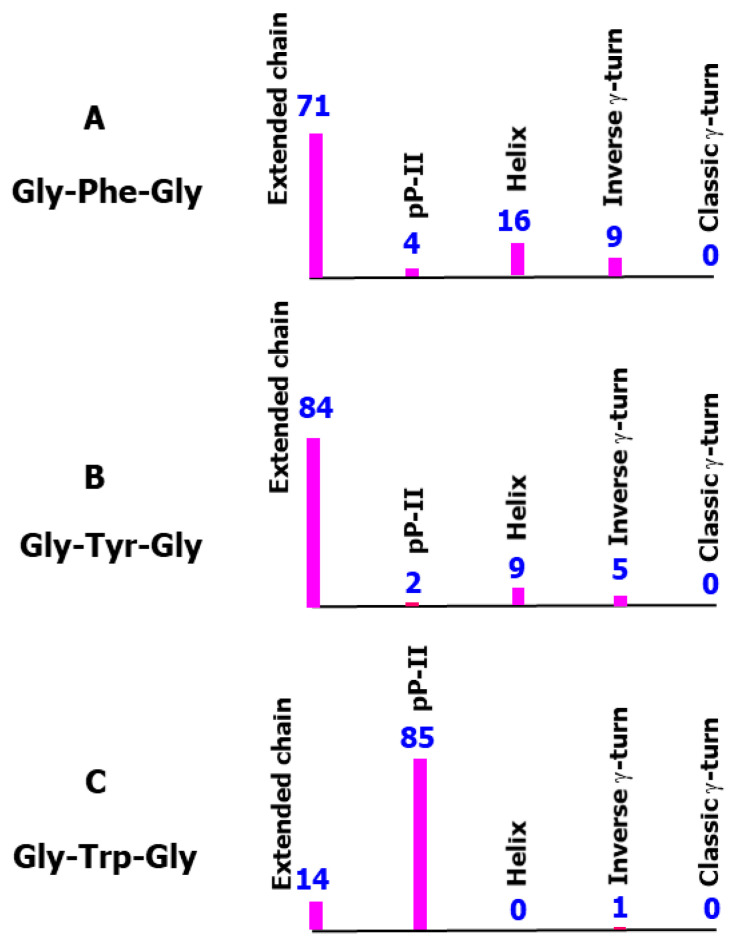
Histograms representing the normalized Boltzmann thermal weights for the five analyzed secondary structures in the aromatic-containing tripeptides. Above each bar, the value of the population, as expressed by thermal weight (in percent), is reported. (**A**) Data obtained from the 15 conformers of Gly-Phe-Gly surrounded by five explicit water molecules and embedded in solvent continuum. (**B**) Data obtained from the 30 conformers of Gly-Tyr-Gly surrounded by six explicit water molecules and embedded in solvent continuum. (**C**) Data obtained from the 30 conformers of Gly-Trp-Gly surrounded by seven explicit water molecules and embedded in solvent continuum.

Supposing that the population of a given secondary structure can be identified by its thermal (Boltzmann) weight, Figure 9 displays the histograms representing the normalized populations (expressed in percent) of each backbone type (extended chains, pP-II, helical, classic and inverse γ-turn), as averaged from all possible aromatic side chain orientations. Quite similar distributions are obtained for Gly-Phe-Gly (Figure 9A) and Gly-Tyr-Gly (Figure 9B), presenting extended chains as major contributions (71% and 84%, respectively), and pP-II, helical, and inverse γ-turn as minor ones. However, subtle differences are found in minor populations in going from one tripeptide to another. In Gly-Trp-Gly, the major population (85%) is ascribed to the pP-II secondary structure, making only one remarkable minor population (14%) assignable to extended chains (Figure 9C).

### 3.2. Assignment of Aromatic Raman Markers

The agreement between the observed and calculated spectra within the middle wavenumber region (Figure 3, Figure 5 and Figure 7) justifies both the consistency of the used theoretical level and the adequacy of the multiconformational approach to calculate the relative energies and Raman intensities of conformers.

Table 1 gives the assignments of aromatic markers derived from the vibrational calculations on the lowest-energy (ΔE = 0) conformers. As can be seen, the three high-wavenumber markers located above 1200 cm^−1^ in Phe (F_1_, F_2_ and F_3_) and Tyr (Y_1_, Y_2_ and Y_3_) originate from quite similar vibrational motions, i.e., basically from the bond stretching motions occurring in phenyl and phenol moieties, respectively. However, substantial differences appear in the markers located below 1200 cm^−1^, among which are the two strong Phe markers (F_4_ and F_5_), as well as the Tyr marker (Y_4_). This effect can be attributed to the contribution of the phenol hydroxyl vibrational motions. Both components of the well-known characteristic Tyr doublet (Y_5_–Y_6_) originate from the fundamental phenol ring vibrational motions. While the higher-wavenumber component of this doublet (Y_5_) results from the bond-stretching motions, the lower-wavenumber one (Y_6_) arises from the out-of-plane bending of the C-H bonds. Nevertheless, the similarity between the vibrational motions responsible for the lowest-wavenumber markers in Phe (F_6_) and Tyr (Y_7_) is to be emphasized. Six out of eight Trp Raman markers (W_1_, …, W_6_) originate from the indole ring’s bond-stretching motions. Particularly, both components of the widely discussed Trp doublet (W_4_–W_5_) are assigned to the indole ring’s fundamental vibrations. The two lowest-wavenumber Trp markers are ascribed to either the in-plane bending (W_7_) or the out-of-plane bending (W_8_) vibrations of the aromatic ring.

**Table 1:** Aromatic Raman markers and their assignments.

Marker	Exp.	Assignment
**Phe**		
F_1_	1605	ν(C_δ1_-C_ε1_); ν(C_δ2_-C_ε2_)
F_2_	1586	ν(C_ε1_-C_ζ_); ν(C_ε2_-C_ζ_); ν(C_γ_-C_δ2_); ν(C_γ_-C_δ1_)
F_3_	1207	ν(C_β_-C_γ_); C_β_-H_2_ rock.;ν(C_γ_-C_δ1_); ν(C_γ_-C_δ2_)
F_4_	1032	ν(C_ε2_-C_ζ_); ν(C_ε1_-C_ζ_); δ(C_ζ_-C_ε1_-H); δ(C_ζ_-C_ε2_-H)
F_5_	1003	δ(C_γ_-C_δ1_-C_ε1_); δ(C_δ1_-C_ε1_-C_ζ_); δ(C_ζ_-C_ε2_-C_δ2_); δ(C_γ_-C_δ2_-C_ε2_)
F_6_	628	δ(C_ζ_-C_ε2_-C_δ2_); δ(C_δ1_-C_ε1_-C_ζ_); δ(C_γ_-C_δ1_-C_ε1_); δ(C_γ_-C_δ2_-C_ε2_)
**Tyr**		
Y_1_	1616	ν(C_δ1_-C_ε1_); ν(C_δ2_-C_ε2_); ν(C_γ_-C_δ1_); ν(C_ε2_-C_ζ_)
Y_2_	1600	ν(C_ε1_-C_ζ_); ν(C_γ_-C_δ2_); ν(C_ε2_-C_ζ_); ν(C_γ_-C_δ1_)
Y_3_	1209	ν(C_β_-C_γ_); C_β_-H_2_ rock.; ν(C_δ1_-C_ε1_); ν(C_δ2_-C_ε2_); ν(C_γ_-C_δ2_); δ(C_δ1_-C_γ_-C_δ2_)
Y_4_	1178	δ(C_γ_-C_δ1_-H); δ(C_ε1_-C_δ1_-H); δ(C_ζ_-O_η_-H); δ(C_ζ_-C_ε2_-H); C_β_-H_2_ twist.; δ(C_δ2_-C_ε2_-H); δ(C_δ1_-C_ε1_-H)
Y_5_	851	ν(C_γ_-C_δ1_); ν(C_β_-C_γ_); ν(C_ε1_-C_ζ_); ν(C_ε2_-C_ζ_); ν(C_γ_-C_δ1_); ν(C_ζ_-O_η_)
Y_6_	828	ω(C_ε2_-H); ω(C_δ2_-H); ω(C_δ1_-H)
Y_7_	643	δ(C_γ_-C_δ1_-C_ε1_); δ(C_γ_-C_δ2_-C_ε2_); δ(C_δ1_-C_ε1_-C_ζ_); δ(C_δ2_-C_ε2_-C_ζ_)
**Trp**		
W_1_	1620	ν(C_ε2_-C_ζ2_); ν(C_ε3_-C_ζ3_)
W_2_	1578	ν(C_δ2_-C_ε3_); ν(C_ζ2_-C_η_); ν(C_ζ3_-C_η_); ν(C_δ2_-C_ε2_)
W_3_	1552	ν(C_γ_-C_δ1_); ν(C_ε2_-C_ζ2_); ν(C_β_-C_γ_)
W_4_	1360	ν(C_γ_-C_δ2_); C_β_-H_2_ twist.
W_5_	1340	ν(C_ζ2_-C_η_); ν(C_ζ3_-C_η_); ν(C_ε2_-C_ζ2_); ν(C_δ2_-C_ε2_)
W_6_	1012	ν(C_ζ3_-C_η_); ν(C_ε3_-C_ζ3_); ν(C_ζ2_-C_η_)
W_7_	880	δ(C_ε2_-N_ε1_-C_δ1_); C_β_-H_2_ wagg.
W_8_	759	ω(C_ζ2_-H); τ(C_ζ2_-C_η_); ω(C_η_-H); ω(C_ζ3_-H); τC_δ2_-C_ε3_); τ(C_ε2_-C_ζ2_)

Marker: notations used for characteristic aromatic Raman lines are reported. (F_i_ = 1, …, 6) for Phe, (Y_i_ = 1, …, 7) for Tyr, and (W_i_ = 1, …, 8) for Trp. Exp.: positions of the main peaks (cm^−1^) taken from the room-temperature aqueous solution Raman spectra of the tripeptides Gly-Phe-Gly (Figure 2A), Gly-Tyr-Gly (Figure 2B), and Gly-Trp-Gly (Figure 2C). Assignments: obtained from the vibrational data of the lowest-energy conformers (ΔE = 0) of the cationic species of Gly-Phe-Gly (Figure 4), Gly-Tyr-Gly (Figure 6), and Gly-Trp-Gly (Figure 8). Based on the potential energy distribution (PED) matrix derived from the vibrational calculations. ν, δ, ω, and τ designate bond stretch, angular bending, out-of-plane bending, and torsion internal coordinates. Rock. (rocking), Wagg. (wagging), and twist. (twisting) reflect the symmetrical coordinates associated with the CH_2_ groups at the β position of the aromatic side chain. See also Figure 1A,B for atomic nomenclature of backbone and side chain.

### 3.3. Wavenumber Dispersion of Aromatic Raman Markers

The average wavenumbers accompanied by their standard deviations calculated across all optimized conformers are reported in Table 2. Average wavenumbers depend on, and reflect, the used theoretical level, whereas standard deviations may bring information on the variation in wavenumbers upon conformational transitions between conformers. Phe markers present the lowest dispersion values (≤5 cm^−1^), whereas larger values are revealed for Tyr markers, among which the most important values are predicted for Y_4_ (17 cm^−1^) and Y_6_ (10 cm^−1^). Comparatively, Trp markers all present a medium wavenumber dispersion, not exceeding 9 cm^−1^.

### 3.4. A New Interpretation for Tyr (Y_5_–Y_6_) and Trp (W_4_–W_5_) Raman Doublets

During past decades, the spectral shape of the Raman doublets arising from the phenol and indole rings was a subject of debate in several reports devoted to the Raman spectroscopy of peptides and proteins [1,2,3,4,5,6,7,8,9,10,11,12,18,41,42]. The Tyr doublet in 1973 [2], and the Trp doublet in 1986 [3], were successively assigned to Fermi-resonance effects. It was suggested that in Tyr, the interaction of a fundamental (planar) mode (Y_5_) with the first overtone of an out-of-plane mode gives rise to the doublet (Y_5_–Y_6_) observed at ~850–830 cm^−1^ [2]. A similar type of interaction was suggested in Trp, but between a planar mode (W_4_) and the additive combination of two out-of-plane modes, resulting in a doublet (W_4_–W_5_) observed at ~1360–1340 cm^−1^ [3]. The reason behind this similarity was in fact due to the simplicity of the used model compounds, and the quite modest computational power available at that time for normal mode calculations. Precisely, aromatic compounds, p-cresol (or methyl-phenol) and skatole (or methyl-indole), were used to interpret the normal modes of Tyr and Trp, respectively. Both molecules were supposed to have a planar (C_s_ symmetry), rendering possible the interpretation of the fundamental modes Y_5_ and W_4_ (Raman active), as well as the out-of-plane modes (IR active) contributing to the formation of the doublets via the aforementioned Fermi-resonance effects. It should be remarked that in C_s_ symmetry, the overtone or the additive combination of IR active modes become Raman-active, justifying the apparition of the Tyr and Trp doublets in Raman spectra. Recent DFT calculations on more sophisticated model compounds [18,41,42], as well as the present one (see Section 4.2 for details), have clearly shown that the components of both doublets, i.e., (Y_5_–Y_6_) and (W_4_–W_5_) arise from the aromatic ring’s fundamental modes, definitely excluding the previously suggested Fermi-resonance-based interpretations [2,3].

**Figure 10:**
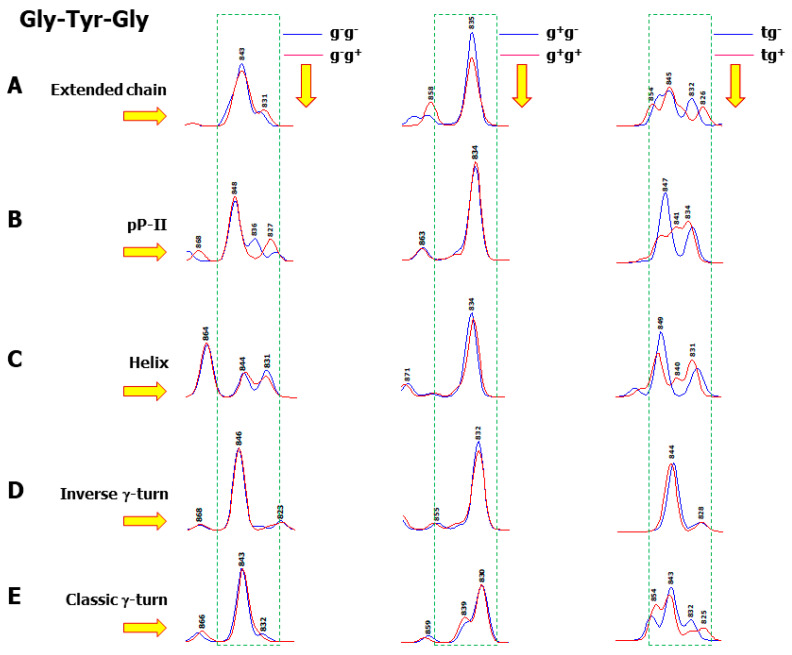
Calculated Raman spectra of the 30 conformers of the cationic species of Gly-Tyr-Gly, displayed in the 900–800 cm^−1^ spectral region. Each conformer is surrounded by six explicit water molecules and embedded in a solvent continuum. Each row represents one of the five different backbone secondary structures: extended chain (**A**), pP-II (**B**), helix (**C**), inverse γ-turn (**D**), and classic γ-turn (**E**). Each column represents two side chain rotamers, with the corresponding spectra drawn in blue and red colours. The frames drawn with broken green lines delimit the spectral region in which the Tyr Raman doublet (Y_5_–Y_6_) is observed.

Furthermore, the observed changes in the relative intensities of the doublets, namely $\rho_{Y}=\frac{I_{850}}{I_{830}}$ (for the Tyr doublet) and $\rho_{W}=\frac{I_{1360}}{I_{1340}}$ (for the Trp doublet), where I is the Raman intensity, have been reported as indicators of the environmental changes in these two aromatic residues. The variation of ρ_Y_ was attributed to H-bonding [2]. It was assumed that when phenol hydroxyl acts as H-bond donor, ρ_Y_ < 1, and in the case where this group becomes a H-bond acceptor, ρ_Y_ > 1. Previous DFT calculations on free amino acid (Tyr) evidenced that the H-bonding on phenol hydroxyl cannot solely explain the observed reversal of ρ_Y_ [18]. The present calculations on the tripeptide Gly-Tyr-Gly give us the opportunity to analyze more completely the conformation and hydration dependence of the Tyr doublet. Figure 10 displays the calculated Raman spectra obtained from the 30 conformers of the tripeptide within the 900–800 cm^−1^ spectral region, drawn by keeping the same scale along the vertical axis for all of them. These spectra are shown as the elements of a 5 × 3 table, where each row corresponds to one of the five considered secondary structures, and each column is relative to the side chain orientations. It is interesting to note that the doublet becomes a singlet or a triplet in certain cases. It can also be perceived that the g^−^ ↔ g^+^ orientation change of the χ_1_ torsion angle plays a key role in ρ_Y_ reversal. More precisely, with a χ_1_(g^−^) orientation, ρ_Y_ < 1, whereas when a χ_1_(g^+^) is adopted, ρ_Y_ > 1. In contrast, with a χ_1_(t) orientation, different situations may appear depending on the χ_2_ orientation, as well as on the backbone secondary structure. 

**Figure 11:**
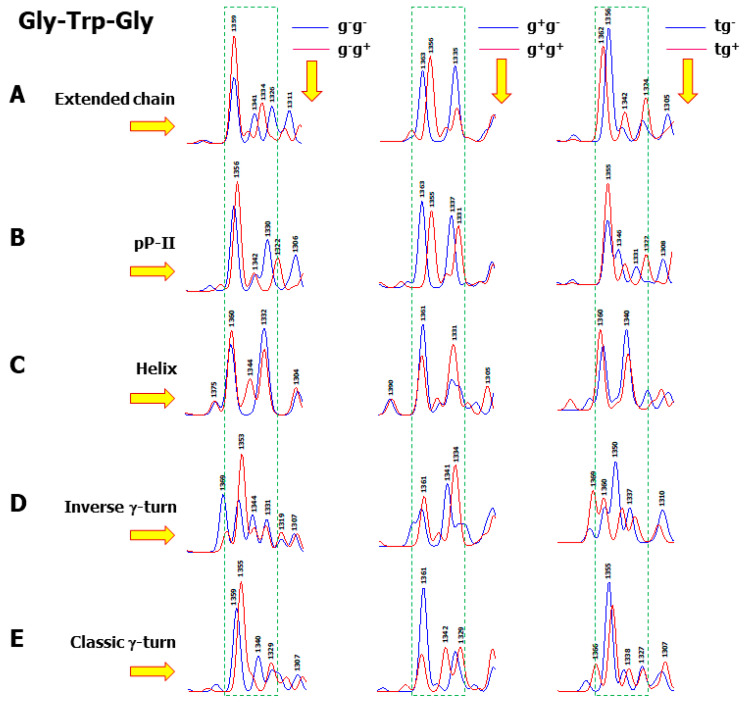
Calculated Raman spectra of the 30 conformers of the cationic species of Gly-Trp-Gly, displayed in the 1400–1300 cm^−1^ spectral region. Each conformer is surrounded by seven explicit water molecules and embedded in a solvent continuum. Each row represents one of the five different backbone secondary structures: extended chain (**A**), pP-II (**B**), helix (**C**), inverse γ-turn (**D**), and classic γ-turn (**E**). Each column represents two side chain rotamers, with the corresponding spectra drawn in blue and red colours. The frames drawn with broken green lines delimit the spectral region in which the Trp Raman doublet (W_4_–W_5_) is observed.

Presumably inspired by the interpretation of ρ_Y_, possible observed ρ_W_ values were suggested, i.e., ρ_W_ > 1 versus ρ_W_ < 1 might be related to the Trp hydrophobic versus hydrophilic environments. Recent preliminary DFT calculations have shown that ρ_W_ reversal mainly depends on the backbone and aromatic side chain conformation [42]. Figure 11 displays the conformational behaviour of the presently calculated Raman spectra obtained from the 30 conformers of Gly-Trp-Gly within the 1400–1300 cm^−1^ spectral region. It can be deduced that in linear backbone structures (extended chain or pP-II), ρ_W_ ≤ 1. The situation becomes different in folded secondary structures (helix, classic and inverse γ-turn), where in many cases the doublet may be transformed into a triplet. These spectra also highlight the combined effects of the side chain χ_1_ and χ_2_ torsion angles on the spectral shape in the analyzed region. For instance, in a helical backbone, while a g^−^g^−^ (or tg^−^) side chain provides ρ_W_ < 1, a g^−^g^+^ (or tg^+^) orientation leads to ρ_W_ > 1. A reverse situation occurs with tg^−^ (ρ_W_ > 1) and tg^+^ (ρ_W_ < 1) side chains. Interestingly, it appears that classic and inverse γ-turn folds can be discriminated, because with the same side chain orientation, different spectral shapes are obtained for these oppositely folded secondary structures.

## 4. Methods

### 4.1. Experimental Details

Tripeptides, with the primary sequence NH_2_-Gly-Xxx-Gly-CONH_2_ (Xxx = Phe, Tyr, Trp), were synthesized at the Institut Pasteur (Paris, France) according to the Fmoc/tBu solid-phase strategy [48] from a Rink amide resin on an ABI 433 synthesizer (Applied Biosystems, Foster City, CA). Details concerning the synthesis procedure and purification were recently reported [41]. The purity control of the final peptides was >98%. The experimental masses were acquired in positive-ion mode, and were consistent with the theoretical isotopic values. Gly-Phe-Gly: expected M + H^+^ 279.1452, observed 279.1441; Gly-Tyr-Gly: expected M + H^+^ 295.1401, observed 295.1383; and Gly-Trp-Gly: expected M + H^+^ 318.1561, observed 318.1539.

Because of the high p*K*_a_ value (~10.5) of the amine terminal group, the cationic form (Figure 1A) remains the major species within a wide pH range (up to ~10) in aqueous solution. Samples containing the tripeptides with trifluoroacetic acid (TFA) as the counterion were prepared at room temperature by dissolving lyophilized powders in water taken from a Millipore filtration system. Upon dissolution, the pH was 6.5 ± 0.1. The final concentration was 20 mM for all tripeptides, i.e., ~5.6 g/L (Gly-Phe-Gly), ~5.9 g/L (Gly-Tyr-Gly), and ~6.3 g/L (Gly-Trp-Gly), leading to a good signal/noise ratio in the Raman spectra.

Then, 50 μL solution samples were placed in a suprasil quartz cell (5 mm path length) and excited by the 488 nm line of an Ar^+^ laser (Spectra Physics), ~200 mW power at the sample. Scattered light at a right angle was analyzed on a T64000 (HORIBA Jobin Yvon, Longjumeau, France) in a single spectrograph configuration with a 1200-groove-per-millimetre holographic grating and a holographic notch filter. Room-temperature Stokes Raman data were collected by means of a liquid nitrogen-cooled charge-coupled device detection system (Spectrum One, Jobin Yvon). The effective spectral slit width was set to ~5 cm^−1^. An average accumulation time of 1200 s (40 scans of 30 s each one) was chosen for recording the reported Raman spectra. Buffer subtraction and smoothing of the observed Raman spectra [41,42,43] was performed by using GRAMS/32 Z.00 (Thermo Galactic, Waltham, MA, United States).

### 4.2. Theoretical Details

Seven torsion angles (ψ_1_, ω_1_, Φ_2_, ψ_2_, ω_2_, Φ_3_, ψ_3_), where subscripts recall residue numbers (Figure 1A), provide together the secondary structure of a cationic tripeptide backbone. Among these angles, ω, defined around the peptide bond (-CO-NH-), remains close to 180°, whereas the (Φ,ψ) pairs define the local conformation of a given residue [49]. To construct the initial conformers of each tripeptide, five distinct secondary structures were considered as follows: β-strand, in which (Φ,ψ) = (−135°,+135°); pP-II with (Φ,ψ) = (−75°,+150°); and α-helix conformation, where (Φ,ψ) = (−60°,−45°). γ-turn folds were supposed to be centred on the middle residue (Phe^2^, Tyr^2^, Trp^2^) (Figure 1B), with the values (Φ_2_,ψ_2_) = (+75°,−65°) and (Φ_2_,ψ_2_) = (−75°,+65°) for the so-called classic and inverse γ-turn, respectively [50,51]. All other backbone torsion angles in a γ-turn conformer, i.e., those relative to Gly^1^ and Gly^3^, were initially set to those relative to a β-strand. Two other torsion angles (χ_1_, χ_2_) detail the aromatic side chain orientation (Figure 1B). Furthermore, [49] χ_1_(N-C_α_-C_β_-C_γ_), around the single bond (C_α_-C_β_), can naturally adopt three privileged orientations, namely gauche^+^ (or g^+^) (60° ± 60°), gauche^−^ (or g^−^) (−60° ± 60°), and trans (or t) (180° ± 60°). In contrast, because of the sp^2^ type of the C_γ_ atom (Figure 1B), χ_2_ defined around C_β_-C_γ_ bond is limited to two privileged orientations, i.e., g^+^(90° ± 90°) and g^−^(−90° ± 90°). Starting with the special case of Gly-Phe-Gly, as C_δ1_ and C_δ2_ atoms located within the phenyl ring are indiscernible, two equivalent definitions become possible for χ_2_, namely χ_2_^(1)^(C_α_-C_β_-C_γ_-C_δ1_) or χ_2_^(2)^(C_α_-C_β_-C_γ_-C_δ2_). One of these two angles is always positive (g^+^), and the other one negative (g^−^), while the absolute value of their difference remains close to 180°. For the sake of clarity, both χ_2_ values were herein reported in Table S1 (Supporting Information), and their orientation was referred to by the particular symbol g^±^. As a result, three distinct Phe^2^ side chain orientations, i.e., g^−^g^±^, g^+^g^±^, and tg^±^, with respect to (χ_1_, χ_2_) angles, were considered. Therefore, with the five considered backbone rotamers and the three plausible side chain orientations, 5 × 3 = 15 initial conformers were prepared for Gly-Phe-Gly. The situation is rather different in Gly-Tyr-Gly, where C_δ1_ and C_δ2_ can be distinguished by the (left/right) orientation of the phenol hydroxyl group (O_η_–H). It should be stated that the two most energetically favourable locations of O_η_–H are within the phenol ring (Figure 1B). To eliminate any ambiguity, both the χ_2_^(1)^ and χ_2_^(2)^ values were reported in Table S2 (Supporting Information), among which χ_2_^(1)^ provides the g^+^ (or g^−^) orientation of χ_2_. Considering the five backbone rotamers and the six side chain orientations (namely g^−^g^−^, g^−^g^+^, g^+^g^−^, g^+^g^+^, tg^−^, and tg^+^), 5 × 6 = 30 initial Gly-Tyr-Gly conformers were prepared. In Gly-Trp-Gly, the definition of χ_2_(C_α_–C_β_–C_γ_–C_δ1_) [49] becomes straightforward because of the asymmetric nature of the indole ring. Nevertheless, to avoid any confusion between the present definition and that previously used by many other authors, i.e., χ^2,1^(C_α_–C_β_–C_γ_–C_δ2_), the values of both torsion angles (χ_2_ and χ^2,1^) were herein reported (Table S3, Supplementary Information). Note that when a g^+^ orientation is assigned to χ_2_, a g^−^ orientation naturally corresponds to χ^2,1^, and inversely. As a result, 5 × 6 = 30 initial conformers were prepared for the Trp-containing tripeptide. In all tripeptides, the initial values given to χ_1_ were as follows: +60° (g^+^), −60° (g^−^), and 180° (t), whereas those assigned to χ_2_ were +90° (g^+^) and -90° (g^−^).

As already mentioned in Section 1, the hydration effects were considered in both implicit and explicit models, which consist of embedding a cluster including the tripeptide in its surrounding water molecules in a polarizable continuum medium, with a relative permittivity corresponding to bulk water (ε_r_ = 78.39). Belonging to the first hydration, all explicit water molecules are capable of forming H-bonds with the polar sites of backbone and aromatic side chains. Starting with the aromatic rings, a pioneering work on benzene [52] revealed that a water molecule can bind to the phenyl ring through the so-called H_w_…***π*** interaction (where H_w_ is a water hydrogen atom interacting with the ***π***-electron cloud of an aromatic ring). More recently, a theoretical work was conducted to find the privileged hydration sites in natural aromatic acids [53]. The most important results derived from this study can be summarized as follows: (i) the H_w_…***π*** interaction was confirmed in the phenyl ring of Phe; (ii) water molecules may bind to the phenol ring in Tyr either through the H_w_…***π*** interaction or by direct H-bonding with its hydroxyl group (O_η_–H); and (iii) in Trp, up to three water molecules can hydrate the indole ring, two of them adopting the H_w_…***π*** interaction type, and a third one by H-bonding to N_ε1_-H (Figure 1B). As far as the backbone of the tripeptides is concerned, inspired by the investigations on amino acids [54] and tripeptides [35,43], a minimal number of explicit water molecules was considered. Precisely, only one water molecule was used to hydrate each backbone terminal group (NH_3_^+^ or CO-NH_2_), while two others were considered to form H-bonds with successive N-H and C=O groups along the backbone chain (Figure 1A). As a result, the hydration number (n) was as follows: n = 5 in Gly-Phe-Gly, n = 6 in Gly-Tyr-Gly, and n = 7 in Gly-Trp-Gly. The initial locations of the hydrating water molecules were those determined by the previous DFT calculations on amino acids [54] and tripeptides [35,43].

Theoretical calculations were performed by means of the Gaussian16 package [55]. The 75 initial conformers for the three tripeptides were submitted to geometry optimization using the DFT approach, M062X hybrid functional, and polarized triple-zeta Gaussian atomic basis set 6-311++G(d,p) [56]. In major cases, optimization was carried out by the default algorithm (eigenvalue following), except in a few cases where a greater number of steps was required to achieve a tight convergence.

Geometry optimization of hydrated conformers was followed by harmonic vibrational calculations. The absence of any imaginary frequency proved the correspondence of an optimized geometry with a local minimum. The energies of the optimized conformers were based on their total energy (E_tot_), where E_tot_ = E_e_ + ΔG, i.e., the sum of electronic energy (E_e_) and free energy correction (ΔG). Therefore, the energy assigned to each conformer includes both enthalpic and entropic contributions. Each optimized conformer was thus characterized by its relative energy (ΔE) compared to that of the lowest-energy conformer, for which the ΔE was set to zero.

Assignment of the vibrational modes was based on the so-called potential energy distribution (PED) [57], as expressed in terms of internal coordinates, i.e., bond stretching (ν), angular bending (δ), out-of-plane bending (ω), and torsion (τ) coordinates. Raman intensities were evaluated based on the calculated Raman activities following the previously reported expression [58]. The calculated Raman intensities, to be compared to experimental spectra, were obtained from the thermal (Boltzmann) average of the spectra derived from different conformers. To scale the raw calculated wavenumbers (ν_calc_), an affine relation of the following form: ν_scal_ = aν_calc_ + b, was used (where ν_scal_ represents scaled wavenumbers and (a,b) are constants). The advantages of this scaling procedure versus a widely used simple linear relation (ν_scal_ = aν_calc_) were detailed in our previous reports [42,43]. See the caption of Table 1 for the (a,b) values determined via linear regression (correlation coefficient > 0.995).

## 5. Concluding Remarks

In the present work, the conformation and hydration dependence of the characteristic aromatic markers were analyzed on the basis of the experimental Raman spectra and the theoretical structural and vibrational data derived from the tripeptides with the primary sequence Gly-Xxx-Gly (where Xxx = Phe, Tyr, Trp). The most striking effect is the structural stability of all considered conformers, especially inverse γ-turn, upon geometry optimization. This is certainly due to the joint use of the M062X functional and explicit backbone hydration, avoiding the opening of folded structures. The influence of the backbone and aromatic ring on the relative energies of conformers has been detailed. Extended chains (β-like) in Gly-Phe-Gly and Gly-Tyr-Gly, and pP-II in Gly-Trp-Gly, were shown to be the most populated conformers in an aqueous environment. The adequacy of the multiconformational analysis of Raman spectra has been highlighted, not only for reproducing the observed data on the tripeptides, but also for predicting the conformation dependence of aromatic markers. On the basis of the theoretical data, a new interpretation was suggested for the characteristic Tyr (~850–830 cm^−1^) and Trp (~1360–1340 cm^−1^) Raman doublets, definitely excluding the previously suggested Fermi-resonance-based assignment of these markers through the consideration of the interactions between the aromatic side chain and its adjacent peptide bonds. 

For the further use of readers, the atomic cartesian coordinates of the low-energy conformers displayed in Figure 4, Figure 6, and Figure 8 are given in Tables S4–S6 (Supporting Information).

## Figures and Tables

**Table 2:** Calculated average values and standard deviations of the wavenumbers of aromatic Raman markers.

Marker Phe ^a^	av ± sd	Marker Tyr ^b^	av ± sd	Marker Trp ^c^	av ± sd
F_1_	1643 ± 2	Y_1_	1621 ± 4	W_1_	1639 ± 2
F_2_	1623 ± 2	Y_2_	1598 ± 7	W_2_	1594 ± 3
F_3_	1219 ± 5	Y_3_	1198 ± 8	W_3_	1560 ± 5
F_4_	1046 ± 3	Y_4_	1164 ± 17	W_4_	1359 ± 4
F_5_	1003 ± 1	Y_5_	848 ± 8	W_5_	1335 ± 6
F_6_	626 ± 4	Y_6_	830 ± 10	W_6_	1013 ± 3
		Y_7_	639 ± 8	W_7_	868 ± 9
				W_8_	760 ± 4

All DFT calculations were performed by means of M062X functional and 6-311++G(d,p) basis set. Average (av.) wavenumbers (cm^−1^) are separated from the corresponding standard deviations (sd.) by “±” symbol. ^a^ Calculated on 15 optimized conformers of Gly-Phe-Gly surrounded by five water molecules and embedded in a solvent continuum. See Table S1 (Supporting Information) for energetic and geometrical parameters of the geometry-optimized conformers. Raw calculated wavenumbers (ν_calc_) were scaled by the affine relation ν_scal_ = aν_calc_ + b, with (a,b) = (0.974, 11.19). ^b^ Calculated on 30 optimized conformers of Gly-Tyr-Gly surrounded by six water molecules and embedded in a solvent continuum. See Table S2 (Supporting Information) for energetic and geometrical parameters of the geometry-optimized conformers. Raw calculated wavenumbers (ν_calc_) were scaled by the affine relation ν_scal_ = aν_calc_ + b, with (a,b) = (0.956, 11.19). ^c^ Calculated on 30 optimized conformers of Gly-Trp-Gly surrounded by seven water molecules and embedded in a solvent continuum. See Table S3 (Supporting Information) for energetic and geometrical parameters of the geometry-optimized conformers. Raw calculated wavenumbers (ν_calc_) were scaled by the affine relation ν_scal_ = aν_calc_ + b, with (a,b) = (0.965, 11.19).

## Data Availability

All data presented in this paper are available upon request.

## Supplementary Materials

The following supporting information can be downloaded at: https://www.mdpi.com/article/10.3390/ijms26083911/s1.
