# Supplementary material for: Influence of Side Chain–Backbone Interactions and Explicit Hydration on Characteristic Aromatic Raman Fingerprints as Analysed in Tripeptides Gly-Xxx-Gly (Xxx = Phe, Tyr, Trp)
Source: Int J Mol Sci. 2025 Apr 21;26(8):3911. doi: 10.3390/ijms26083911 (PMC12027947; doi:10.3390/ijms26083911)
Supplement: Supplementary file 1 [file ijms-26-03911-s001.zip › ijms-3558104-supplementary.pdf]

Supplementary Material

Influence of Side Chain-Backbone Interactions and Explicit Hydration on Characteristic Aromatic Raman Fingerprints as Analysed in Tripeptides Gly-Xxx-Gly (Xxx=Phe, Tyr, Trp)

Belén Hernández[1], Yves-Marie Coïc[2], Sergei G. Kruglik[3,4], Santiago Sanchez-Cortes[5], Mahmoud Ghomi[5,*]

[1]LVTS, INSERM U1148. 74 rue Marcel Cachin. 93017 Bobigny Cédex France

[2]Institut Pasteur, Université Paris Cité, CNRS UMR 3523, Unité de Chimie des Biomolécules, F-75015 Paris, France

[3]Sorbonne Université, CNRS, Inserm, Institut de Biologie Paris-Seine, IBPS, Laboratoire Jean Perrin, LJP, F-75005 Paris, France

[4]Université Paris Cité, CNRS, Inserm, Laboratoire de Nanomédecine, Biologie Extracellulaire, Intégratome et Innovations en santé, NABI, F-75006 Paris, France

[5]Instituto de Estructura de la Materia – Consejo Superior de Investigaciones Ciebtificas (IEM-CSIC), 28006 Madrid, Spain

*Corresponding author: M. Ghomi, E-mail: mahmoud.ghomi@univ-paris13.fr

Content

<u>This document contains 12 pages</u>

Table S1 Conformational angles and relative energies obtained by means of M062X functional on cationic species of Gly-Phe-Gly conformers in presence of 5 water molecules, all embedded in a solvent continuum **Page 2**

Table S2 Conformational angles and relative energies obtained by means of M062X functional on cationic species of Gly-Tyr-Gly conformers in presence of 6 water molecules, all embedded in a solvent continuum **Page 3**

Table S3 Conformational angles and relative energies obtained by means of M062X functional on cationic species of Gly-Trp-Gly conformers in presence of 7 water molecules, all embedded in a solvent continuum **Page 4**

Table S4 Atomic cartesian coordinates of the 7 lowest energy conformers of cationic Gly-Phe-Gly+5 water molecule **Page 5**

Table S5 Atomic cartesian coordinates of the 3 lowest energy conformers of cationic Gly-Tyr-Gly+6 water molecules **Page 9**

Table S6 Atomic cartesian coordinates of the 2 lowest energy conformers of cationic Gly-Trp-Gly+7 water molecules **Page 11**

Table S1 Conformational angles and relative energies obtained by means of M062X functional on cationic species of Gly-Phe-Gly conformers in presence of 5 water molecules, all embedded in a solvent continuum

**Conformers**	**Backbone**							**Side-chain**		**d_{HB}**	**ΔE**
	ψ_1	ω_1	ϕ_2	ψ_2	ω_2	ϕ_3	ψ_3	χ_1	$\chi_2^{(1)}/\chi_2^{(2)}$		
Extended chain/g$^-$g$^\pm$	+161.5	-179.1	-119.0	+126.7	+164.6	-75.6	+164.7	-63.9	+112.7/-68.1		0.0
Extended chain/g$^+$g$^\pm$	+175.7	-175.4	-153.5	+159.3	+176.8	-70.9	+145.0	+53.4	+83.2/-93.9		+1.68
Extended chain/tg$^\pm$	+179.5	-177.1	-160.3	+145.9	+172.7	-162.7	+176.4	-164.2	+92.1/-85.6		+0.39
Helix/g$^-$g$^\pm$	+158.9	+179.5	-74.1	-32.8	-178.3	-69.2	-17.1	-57.6	+105.7/-73.0	2.08	+3.15
Helix/g$^+$g$^\pm$	-177.8	+177.9	-60.6	-22.0	180.0	-69.9	-20.1	+58.8	+79.2/-98.8	2.12	+0.65
Helix/tg$^\pm$	+166.1	+178.7	-57.7	-36.4	-177.8	-90.0	+2.9	-173.7	+92.7/-87.1	2.09	+3.51
Inverse γ-turn/g$^-$g$^\pm$	-160.5	-171.5	-87.0	+64.1	-178.7	+171.9	+160.4	-55.7	+129.1/-51.8	2.06	+1.14
Inverse γ-turn/g$^+$g$^\pm$	-138.3	-177.0	- 82.4	+51.3	+179.2	+70.1	-137.6	+43.9	+79.2/-100.0	2.02	+1.78
Inverse γ-turn/tg$^\pm$	+162.4	+178.6	-84.9	+81.2	-177.2	+172.6	+179.5	-179.0	+55.0/-125.5	2.27	+4.01
pP-II/g$^-$g$^\pm$	-172.4	+171.5	-65.5	+153.0	+168.7	-67.3	+168.3	-59.8	+111.0/-68.8		+1.74
pP-II/g$^+$g$^\pm$	-170.9	+169.7	-64.0	+154.8	+176.7	-69.0	+158.5	+52.6	+81.0/-97.2		+2.36
pP-II/tg$^\pm$	-176.2	+171.5	-65.0	+152.7	+176.4	-62.1	+158.7	-157.0	+85.9/-93.1		+3.04
Classic γ-turn/g$^-$g$^\pm$	+167.8	+161.1	+78.4	-37.7	-176.6	+174.1	+178.5	-54.0	+101.3/-74.9	2.01	+4.37
Classic γ-turn/g$^+$g$^\pm$	+162.9	+164.4	+57.8	-15.2	-179.1	-179.0	+174.0	+66.8	+78.6/-100.5	1.91	+7.66
Classic γ-turn/tg$^\pm$	+163.3	+171.7	+77.7	-58.5	-178.0	+176.3	+177.9	-168.9	+93.9/-84.6	2.01	+3.38

Conformational angles are expressed in degrees (see Fig. 1, main text, for their definition). Relative energies (ΔE) are reported in kcal/mol. Graphical representation of the 7 lowest energy conformers are displayed in Fig. 4 (main text). d_{HB} represents the intramolecular hydrogen bond length (in Å) formed in helical, Inverse and Classic γ-turn conformers.

Table S2 Conformational angles and relative energies obtained by means of M062X functional on cationic species of Gly-Tyr-Gly conformers in presence of 6 water molecules, all embedded in a solvent continuum

**Conformers**	**Backbone**							**Side-chain**		**d_{HB}**	**ΔE**
	ψ_1	ω_1	ϕ_2	ψ_2	ω_2	ϕ_3	ψ_3	χ_1	$\chi_2^{(1)}/\chi_2^{(2)}$		
Extended chain/g^-g^-	+159.5	-179.5	-118.3	+129.0	+163.4	-71.8	+160.9	-64.5	-64.1/+117.1		+2.40
Extended chain/g^-g^+	+161.3	-179.4	-118.9	+127.2	+164.6	-74.7	+164.3	-63.4	+115.2/-65.9		+2.96
Extended chain/g^+g^-	+177.1	-174.9	-153.1	+158.1	+176.2	-69.7	+145.8	+53.4	-93.9/+83.7		+2.38
Extended chain/g^+g^+	+176.1	-175.1	-155.8	+162.0	+176.4	-178.4	+173.6	+55.4	+82.0/-95.0		+3.84
Extended chain/tg^-	+166.8	-179.5	-155.6	+137.6	+172.9	-127.1	+146.5	-171.9	-82.1/+95.8		0.
Extended chain/tg^+	+173.4	-178.4	-148.6	+135.4	+175.9	-83.6	+83.9	-178.8	+85.9/-91.9		+4.03
Helix/g^-g^-	+158.3	-179.8	-59.1	-32.9	-177.8	-70.2	- 16.2	-55.2	-72.2/+105.4	2.10	+4.48
Helix/g^-g^+	+158.8	+178.9	-57.7	-34.0	-177.7	-69.8	-16.1	-56.1	+104.6/-73.8	2.08	+3.91
Helix/g^+g^-	-175.4	+178.1	-59.6	-22.7	+179.4	-69.1	-20.4	+56.6	-98.2/+80.1	2.07	+2.22
Helix/g^+g^+	-179.2	+178.2	-59.2	-23.7	-179.1	-70.4	-18.7	+58.5	+80.2/-98.0	2.10	+1.61
Helix/tg^-	+166.9	+174.0	-57.7	-41.3	-176.8	-82.8	-2.0	-177.7	-83.3/+95.9	2.24	+4.30
Helix/tg^+	+167.1	+172.8	-57.5	-40.4	-177.6	-86.8	+3.0	-172.1	+94.4/-84.6	2.21	+3.69
Inverse γ-turn/g^-g^-	-160.5	-169.5	-88.0	+63.9	-178.0	+171.7	+159.3	-51.1	-33.6/+146.2	2.05	+2.72
Inverse γ-turn/g^-g^+	-162.0	-169.1	-88.7	+63.3	-177.8	+169.9	+163.4	-49.8	+143.5/-53.5	2.06	+1.92
Inverse γ-turn/g^+g^-	-137.6	-178.2	-81.7	+52.9	+177.7	+68.7	-137.6	+42.3	-100.6/+78.5	2.00	+2.73
Inverse γ-turn/g^+g^+	-140.0	-176.9	-82.0	+51.8	+178.4	+71.7	-136.8	+43.3	+78.2/-100.9	2.01	+3.36
Inverse γ-turn/tg^-	+163.9	+179.3	-84.1	+83.1	-177.3	+176.8	+177.0	-179.5	-123.2/+57.5	2.27	+4.92
Inverse γ-turn/tg^+	+166.1	+179.6	-84.1	+81.4	-177.7	+175.9	+178.3	-178.8	+57.3/-123.4	2.24	+5.47
pP-II/g^-g^-	-173.5	+171.5	-66.1	+153.3	+170.5	-66.1	+162.9	-59.7	-69.5/+109.5		+3.96
pP-II/g^-g^+	-173.7	+172.1	-66.4	+155.0	+170.2	-64.4	+161.8	-59.6	+110.5/-68.7		+3.68
pP-II/g^+g^-	-172.7	+169.9	-64.7	+155.5	+178.2	-69.6	+157.8	+54.2	-97.7/+80.5		+2.33
pP-II/g^+g^+	-175.1	+169.4	-65.1	+155.8	+178.2	-68.9	+154.8	+54.0	+81.0/-97.3		+3.48
pP-II/tg^-	-175.2	+171.4	-62.8	+150.1	+179.4	-62.9	+156.0	-159.5	-89.4/+89.0		+4.27
pP-II/tg^+	-174.1	+172.4	-65.5	+153.0	+176.8	-64.8	+163.3	-157.1	+86.4/-92.4		+4.30
Classic γ-turn/g^-g^-	+167.8	+159.6	+78.9	-30.9	-177.5	+179.4	+175.0	-53.3	-74.0/+102.3	2.07	+5.88
Classic γ-turn/g^-g^+	+164.0	+159.7	+79.3	-31.9	-178.0	+177.9	+176.0	-53.8	+102.0/-74.2	2.06	+5.91
Classic γ-turn/g^+g^-	+163.1	+165.2	+58.1	-16.6	-178.4	+178.5	+175.1	+67.7	-100.7/+78.0	1.91	+8.43
Classic γ-turn/g^+g^+	+162.5	+164.9	+57.3	-15.8	-178.8	+179.5	+174.1	+66.6	+78.1/-100.4	1.90	+8.07
Classic γ-turn/tg^-	+162.2	+172.0	+76.7	-60.1	-177.3	+173.3	-179.0	-170.5	-83.7/+94.8	2.00	+5.82
Classic γ-turn/tg^+	+161.7	+171.2	+77.3	-59.3	-177.3	+174.4	-179.2	-168.5	+92.4/-85.9	2.00	+5.33

Conformational angles are expressed in degrees (see Fig. 1, main text, for their definition). Relative energies (ΔE) are reported in kcal/mol. Graphical representation of conformers are displayed in Fig. 6 (main text). d_{HB} represents the intramolecular hydrogen bond length (in Å) formed in helical, Inverse and Classic γ-turn conformers.

Table S3 Conformational angles and relative energies obtained by means of M062X functional on cationic species of Gly-Trp-Gly conformers in presence of 7 water molecules, all embedded in a solvent continuum

**Conformers**	**Backbone**							**Side-chain**		**d_{HB}**	**ΔE**
	ψ_1	ω_1	ϕ_2	ψ_2	ω_2	ϕ_3	ψ_3	χ_1	χ_2/ $\chi^{2,1}$		
pP-II/g$^-$g$^-$	+179.8	-175.3	-136.1	+134.7	+162.9	-72.8	+160.4	-67.3	-93.8/+84.5		+4.92
pP-II/g$^-$g$^+$	+135.8	-164.7	-150.7	+155.7	+169.5	-64.5	+161.1	-49.5	+98.8/-74.3		+9.39
pP-II/g$^+$g$^-$	-168.7	+170.1	-64.3	+158.8	+174.9	-124.2	+166.9	+58.4	-93.9/+82.5		+5.06
pP-II/g$^+$g$^+$	-158.4	+172.2	-61.8	+157.5	+177.7	-64.7	+148.8	+55.9	+83.4/-91.4		0.
pP-II/tg$^-$	-171.0	+170.3	-62.7	+135.0	+177.9	-86.1	+79.1	+171.0	-99.2/+70.9		+4.77
pP-II/tg$^+$	+178.0	+176.3	-94.1	+134.6	+157.1	-127.4	+155.6	+178.4	+88.7/-90.6		+5.95
Extended chain/g$^-$g$^-$	+141.5	-171.8	-146.7	+133.4	+165.7	-75.4	+160.6	-58.0	-86.0/+85.4		+6.26
Extended chain/g$^-$g$^+$	+167.3	-174.1	-135.6	+133.1	+177.7	+177.8	-179.6	-67.7	+94.4/-76.1		+7.47
Extended chain/g$^+$g$^-$	+165.0	-175.8	-156.0	+161.5	+171.6	-137.4	+174.3	+60.0	-88.9/+84.7		+5,42
Extended chain/g$^+$g$^+$	+155.3	-173.9	-167.1	+157.8	+169.3	-65.6	+165.2	+45.2	+80.5/-92.6		+4.62
Extended chain/tg$^-$	+165.3	+176.2	-113.3	+132.0	+168.3	-114.4	+140.2	+177.5	-97.6/+73.9		+2.65
Extended chain/tg$^+$	-161.4	+167.7	-54.6	+145.6	+171.4	-138.8	+168.9	-178.5	+95.8/-81.4		+1.12
Helix/g$^-$g$^-$	-154.8	-170.5	-65.4	-22.0	+178.6	-83.6	-5.8	-62.2	-79.0/+96.1	2.04	+4.56
Helix/g$^-$g$^+$	-157.3	-170.6	-65.7	-22.8	+179.0	-83.4	-6.1	-54.6	+111.5/-64.4	2.04	+3.69
Helix/g$^+$g$^-$	-173.9	+178.3	-60.0	-24.3	-178.4	-75.5	-10.3	+58.0	-97.1/+79.4	2.12	+5.65
Helix/g$^+$g$^+$	-171.0	180.0	-61.8	-23.2	-179.0	-79.3	-79.3	-6.6	+86.4/-87.1	2.11	+5.97
Helix/tg$^-$	-153.5	-171.8	-59.4	-33.6	-178.4	-77.3	-7.1	180.0	-101.8/+72.5	2.02	+3.92
Helix/tg$^+$	+152.0	+175.1	-58.0	-39.6	-174.7	-76.4	-5.6	+173.8	+86.2/-88.9	2.27	+7.75
Inverse γ-turn/g$^-$g$^-$	-159.4	-174.7	-88.8	+68.6	- 178.1	+169.6	+159.1	-64.6	-88.7/+89.1	2.17	+5.78
Inverse γ-turn/g$^-$g$^+$	-161.1	-172.5	-86.6	+65.0	-178.8	+171.4	+159.8	-58.5	+111.9/-65.2	2.07	+4.16
Inverse γ-turn/g$^+$g$^-$	-153.5	+174.7	-92.7	+66.4	-174.7	+61.1	-140.3	+53.4	-74.9/+109.8	2.37	+5.33
Inverse γ-turn/g$^+$g$^+$	-174.2	-174.6	-90.2	+53.8	+179.8	+177.8	-177.1	+47.8	+84.1/-93.1	2.20	+8.79
Inverse γ-turn/tg$^-$	-159.7	-174.9	-87.2	+78.4	-177.1	+159.1	+158.9	-171.3	-108.8/+70.0	2.20	+5.05
Inverse γ-turn/tg$^+$	-158.2	+174.5	-83.9	+83.9	-179.6	+55.4	-132.7	+176.8	+68.5/-106.9	2.32	+6.15
Classic γ-turn/g$^-$g$^-$	+164.8	+162.0	+70.4	-41.1	+179.3	-169.4	-176.9	-50.5	-70.9/+99.5	1.90	+6.64
Classic γ-turn/g$^-$g$^+$	+166.9	+156.0	+82.1	-40.3	-176.7	+175.1	+176.5	-43.5	+110.6/-59.6	2.00	+8.98
Classic γ-turn/g$^+$g$^-$	+165.6	+165.6	+57.5	-28.7	- 171.5	+153.1	-171.1	+60.9	-96.4/+76.0	1.82	+10.72
Classic γ-turn/g$^+$g$^+$	-152.5	+168.3	+56.7	-45.0	-177.8	-85.7	+126.2	+49.1	+87.1/-85.7	1.78	+10.30
Classic γ-turn/tg$^-$	+165.4	+171.8	+76.2	-60.4	-176.2	+172.9	+177.9	-175.1	-102.4/+71.1	2.00	+8.78
Classic γ-turn/tg$^+$	-168.2	+176.5	+75.4	-57.2	-171.2	+155.0	-178.6	-174.9	+88.1/-88.1	1.99	+7.92

Conformational angles are expressed in degrees (see Fig. 1, main text, for their definition). Relative energies (ΔE) are reported in kcal/mol. Graphical representation of conformers are displayed in Fig. 8 (main text). d_{HB} represents the intramolecular hydrogen bond length (in Å) formed in helical, Inverse and Classic γ-turn conformers.

Table S4 Atomic cartesian coordinates of the 7 lowest energy conformers of cationic Gly-Phe-Gly+5 water molecules*

Extended chain with $g^-g^\pm$ side chain			ΔE=0	Extended chain with $tg^\pm$ side chain			ΔE=0.39
C	-3.03259100	-2.91181800	0.33039900	C	-4.88207600	-0.61274400	0.20020900
C	-1.98645100	-2.06223100	-0.38239800	C	-3.50088000	-0.09313000	-0.18515300
O	-2.06955400	-1.88477500	-1.59140600	O	-3.37750300	0.78292700	-1.03478800
H	-3.33615400	-2.47590700	1.28037600	H	-5.07316000	-0.45697200	1.26117800
N	-1.02753000	-1.57307200	0.41099300	N	-2.49018000	-0.66907100	0.46859700
C	0.03933600	-0.72823400	-0.09635200	C	-1.12070300	-0.23753500	0.25787100
C	1.39844900	-1.38402700	0.13575300	C	-0.19914300	-1.35858200	0.72914300
O	1.75665400	-1.73996400	1.25588000	O	-0.47900500	-2.03505300	1.71901200
C	0.03719800	0.63563000	0.61306100	C	-0.82853900	1.06009700	1.05632400
H	-1.06120400	-1.73151100	1.41941200	H	-2.68976500	-1.33628000	1.21552200
H	-0.13001400	-0.58790600	-1.16381400	H	-0.97472900	-0.04508600	-0.80780500
H	0.15360400	0.47381600	1.68758400	H	-1.68627100	1.71958000	0.91114300
N	2.17731500	-1.50598300	-0.95247100	N	0.93453600	-1.50643300	0.04021400
C	3.58212300	-1.79404100	-0.80652800	C	1.94927400	-2.42921000	0.49352100
O	3.85900800	0.57395400	-0.44978700	O	3.42013300	-1.27262700	-1.02789300
H	1.87638100	-1.03121800	-1.79639300	H	1.11915700	-0.89942400	-0.75859300
H	3.71977200	-2.60060800	-0.08640600	H	2.05602700	-2.35350600	1.57928000
N	-4.21880600	-2.98451900	-0.55736000	N	-5.89303500	0.12602300	-0.59493500
H	-3.93937400	-3.30237000	-1.48925400	H	-5.72368600	-0.04037800	-1.61382800
H	-4.93511300	-3.61200900	-0.19148800	H	-6.84083200	-0.16665700	-0.35494800
C	4.35468800	-0.55275900	-0.35463100	C	3.28797200	-2.14188600	-0.16366600
N	5.58507600	-0.76917400	0.11303200	N	4.30046500	-2.90429800	0.25733000
H	5.96126800	-1.70077100	0.19540900	H	4.17101700	-3.60807200	0.96736700
H	6.15889600	0.02632700	0.37643000	H	5.21858900	-2.77184800	-0.15587200
H	0.90676000	1.19854800	0.26219700	H	-0.77319300	0.80794100	2.11879700
H	-4.60125000	-2.02579100	-0.66428600	H	-5.81819100	1.13287500	-0.43542100
C	-1.22413300	1.41168300	0.33168800	C	0.42332200	1.75690600	0.59129900
C	-2.15268600	1.67100100	1.33981200	C	0.33996900	2.74814100	-0.39115200
C	-1.46983900	1.89942300	-0.95737600	C	1.68162400	1.40591100	1.08538600
C	-3.29482400	2.42939800	1.07694300	C	1.48902700	3.37145600	-0.87500500
H	-1.97531900	1.29061000	2.34073100	H	-0.63324100	3.03246700	-0.77919600
C	-2.61094400	2.65194100	-1.22386500	C	2.83406800	2.01814700	0.59637100
H	-0.74286700	1.70560200	-1.74090400	H	1.76334600	0.64676800	1.85746400
C	-3.52426500	2.92502900	-0.20378800	C	2.74117900	3.00117000	-0.38631900
H	-4.00071800	2.63342300	1.87376300	H	1.40534700	4.14315100	-1.63140200
H	-2.78389800	3.03440300	-2.22303200	H	3.80320900	1.72737100	0.98430300
H	-4.40669600	3.51920400	-0.40943400	H	3.63632800	3.48041700	-0.76363100
H	3.98649700	-2.11922500	-1.76646000	H	-4.97495500	-1.67379600	-0.02751500
H	-2.64907100	-3.91828300	0.49505900	H	1.66840400	-3.46348200	0.26990200
H	-1.06609300	4.15240700	1.51243500	O	-4.90465800	-0.41725900	-3.08337400
O	-0.65195300	4.71972900	0.85416900	H	-5.24617000	-0.36846400	-3.98131600
H	-0.88770500	4.30539800	0.01711900	H	-4.11644400	0.13912700	-3.04960800
H	2.48207100	1.05542600	-1.66102600	O	1.24292000	4.58687600	2.36871300
O	1.68520300	1.02214500	-2.21783000	H	0.79324200	4.64428900	1.51941500
H	1.85032300	1.61399800	-2.95687900	H	1.73470500	3.76064300	2.30771600
H	5.11174400	1.88860800	0.17378200	H	5.26517000	-1.20333600	-1.60990100
O	5.95838100	2.07819100	0.60943600	O	6.17939000	-1.49806300	-1.47320100
H	6.32572600	2.84302000	0.15900700	H	6.53707100	-1.66894400	-2.34848700
H	-4.59764000	0.48663400	-0.15378600	H	1.46565700	1.19916400	-2.06083500
O	-4.33397900	-0.24795400	-0.71826100	O	1.47230000	0.26202500	-2.28709900
H	-3.54953200	0.05525700	-1.19266700	H	2.35332800	-0.05659300	-2.03949700
O	-0.26770400	-1.93660400	3.15404900	O	-3.06788700	-2.48330600	2.68050100
H	0.59895000	-1.84591900	2.72086800	H	-3.46806700	-3.35683400	2.65260700
H	-0.26654800	-1.32568700	3.89597100	H	-2.11047900	-2.62426400	2.62677500
**Helical with $g^+g^\pm$ side chain**			**ΔE=+0.65**	**Inverse γ-turn with $g^-g^\pm$ side chain**			**ΔE=+1.14**
N	2.00565000	3.91678200	-1.04128900	C	-0.16745500	-3.03901000	0.56246200
H	2.54119800	3.16378900	-1.47841500	C	0.04092700	-1.70177500	-0.13241900

C	0.64103400	3.46334100	-0.68205900	O	0.73973100	-1.63816200	-1.14103700
H	2.51588700	4.20296000	-0.17200000	H	-1.12295900	-3.47033300	0.26466000
H	1.97212400	4.70221400	-1.69261800	N	-0.58565100	-0.66153000	0.42235800
C	0.76161800	2.27321900	0.25480400	C	-0.63387300	0.64448200	-0.22531700
H	0.12295200	4.28787500	-0.19358600	C	0.60426500	1.47866400	0.14352900
O	1.85943700	1.85689000	0.61109300	O	0.51753700	2.52520800	0.77951100
N	-0.39923000	1.72240100	0.63136000	C	-1.91805400	1.37555600	0.15767000
H	-1.25488200	2.04064700	0.19191400	H	-1.16075300	-0.82901200	1.25044600
C	-0.43521600	0.55023700	1.48907900	H	-0.59451700	0.47178500	-1.30449400
H	0.11739800	0.76469600	2.40617500	H	-1.88672500	1.61614700	1.22281900
C	0.26627600	-0.65979000	0.86825900	N	1.77168700	0.97798100	-0.28329300
O	0.66268500	-1.56482200	1.59697100	C	3.01892200	1.64280800	0.01543100
N	0.40906700	-0.67640500	-0.46611700	O	4.03351700	-0.45121600	-0.56714900
H	-0.06516200	0.01733700	-1.04336300	H	1.78615000	0.10080300	-0.79760900
C	1.05495100	-1.79796400	-1.10404500	H	3.07528200	2.61678800	-0.47796000
C	2.55299200	-1.88187000	-0.83236800	N	0.93386200	-3.93170200	0.12665400
H	0.61522300	-2.74571600	-0.78459500	H	1.82479300	-3.51613700	0.47588000
O	3.12835200	-2.95986000	-1.02177900	H	0.82348400	-4.88119800	0.48206200
N	3.17539800	-0.77498800	-0.42746600	C	4.18161900	0.76736400	-0.40960300
H	2.66953600	0.07535600	-0.20913700	N	5.34980500	1.38236200	-0.57550700
H	4.17259600	-0.82566600	-0.24515700	H	5.43891600	2.38077800	-0.46757300
C	-1.89000300	0.19962400	1.85707900	H	6.16768800	0.82679900	-0.81092600
H	-1.85665700	-0.67617900	2.50768600	H	-1.93408700	2.32783600	-0.37859700
H	-2.30230600	1.02921100	2.43446200	H	0.98279000	-3.95928700	-0.89473100
C	-2.74534600	-0.07294500	0.64313700	C	-3.16459200	0.58000000	-0.15355700
C	-2.67924900	-1.31007500	-0.00793200	C	-4.13992200	0.38800600	0.82796100
C	-3.58597800	0.91449900	0.12332000	C	-3.37916700	0.03609700	-1.42339700
C	-3.41624600	-1.54628500	-1.16344400	C	-5.30180000	-0.33363800	0.55175100
H	-2.04874300	-2.09605400	0.39748500	H	-3.99135400	0.81263300	1.81604400
C	-4.33021200	0.68039200	-1.03456600	C	-4.53540700	-0.68716300	-1.70182100
H	-3.67377800	1.87001100	0.63182200	H	-2.64278500	0.18161900	-2.20727400
C	-4.24013500	-0.54824400	-1.68423500	C	-5.50021300	-0.87657300	-0.71425700
H	-3.35244800	-2.50956700	-1.65526600	H	-6.04863200	-0.46829100	1.32569500
H	-4.97945500	1.45583200	-1.42314900	H	-4.68407000	-1.10180600	-2.69208300
H	-4.81671700	-0.73315700	-2.58238500	H	-6.40033000	-1.43831600	-0.93270600
H	0.09738500	3.17542700	-1.58207500	H	-0.13881400	-2.94248800	1.64512600
H	0.92255800	-1.71192600	-2.18352300	H	3.10857900	1.82026500	1.09312700
H	3.07289000	3.42889000	1.79164800	H	2.00686500	5.20891700	1.85713100
O	2.94900000	4.33288300	1.47847800	O	2.45869200	4.43653100	1.50847500
H	3.67570400	4.85284500	1.83520000	H	1.75236700	3.81260100	1.27274500
H	-5.64254400	-0.61789700	0.91104200	H	-5.48248200	2.15872800	-0.50499500
O	-5.82020100	-1.44382300	1.37380400	O	-5.59680800	2.62864700	-1.33839200
H	-5.04049200	-1.97911600	1.19548000	H	-5.16657600	2.05536400	-1.98085400
O	5.70703600	-2.26714300	-0.25726800	O	2.68806200	-2.15729800	1.16082400
H	6.45666700	-2.41279900	-0.83997800	H	3.13944400	-1.55409800	0.54121800
H	4.96769700	-2.77319600	-0.63479100	H	3.26636200	-2.24372900	1.92430100
H	1.35074700	-3.34853000	1.42613300	O	6.64557000	-1.08229900	-1.36864800
O	1.71297900	-4.23051200	1.25166000	H	5.73017100	-1.25727600	-1.10048400
H	2.27912100	-4.10213600	0.48027300	H	7.17857400	-1.77526200	-0.97006600
H	-0.91512700	1.10909700	-3.14280400	H	-3.20712200	-1.65939000	2.17085100
O	-1.14820300	1.10514500	-2.20807600	O	-2.29037300	-1.73043500	2.46001800
H	-2.07316800	0.82633900	-2.18315000	H	-2.30708000	-1.56093100	3.40735500

**Extended chain with $g^+g^\pm$ side chain**			**ΔE=+1.68**	**pP-II with $g^-g^\pm$ side chain**			**ΔE=+1.74**
C	3.98519300	-1.07439700	-1.27907000	C	2.66047400	-2.64284500	-1.27638500
C	3.02110000	-1.34286700	-0.12861700	C	1.76568600	-2.01733700	-0.21615800
O	3.44183900	-1.69422700	0.96796700	O	1.54951900	-2.58348200	0.84823500
H	3.85196700	-0.06708600	-1.67334200	H	3.59151900	-2.08327200	-1.36445400
N	1.73394200	-1.17096700	-0.43965500	N	1.26552400	-0.81720600	-0.54177200
C	0.69384700	-1.28857300	0.56360900	C	0.25751500	-0.19967100	0.29707600
C	-0.60852400	-1.69850600	-0.12119400	C	-1.03309500	-1.02561000	0.26921600
O	-0.80969100	-1.52570800	-1.31993800	O	-1.34897900	-1.69973500	-0.70952300

C	0.52737300	0.03028700	1.36365900	C	-0.05375300	1.21746700	-0.20891000
H	1.49002200	-0.75155100	-1.33899400	H	1.30328400	-0.54174500	-1.51842200
H	0.98756300	-2.07677700	1.25925200	H	0.63158600	-0.14850600	1.32173800
H	-0.30429100	-0.08375100	2.06381400	H	-0.46793900	1.15107000	-1.21990200
N	-1.52801600	-2.25892700	0.68195200	N	-1.79950100	-0.92036000	1.36156500
C	-2.82306100	-2.62110900	0.15462800	C	-3.13727200	-1.45054000	1.35091200
O	-3.63350200	-0.40102800	0.57654000	O	-3.70964400	0.40749400	-0.07460600
H	-1.41056000	-2.23214000	1.68852400	H	-1.51879200	-0.26853800	2.09185000
H	-2.69918000	-3.23955700	-0.73500000	H	-3.12364100	-2.50178000	1.05623200
N	5.37088400	-1.20065800	-0.76596500	N	2.98901700	-4.03013900	-0.86550100
H	5.52484700	-2.13001300	-0.36814100	H	2.14333400	-4.59741300	-0.77620900
H	6.05595800	-1.05055900	-1.50787800	H	3.59623800	-4.47943200	-1.55207800
C	-3.67593900	-1.39503400	-0.15801700	C	-4.04844800	-0.67251300	0.39887600
N	-4.47973600	-1.47817200	-1.21655100	N	-5.23330800	-1.25029900	0.14940800
H	-4.46824200	-2.28154800	-1.82472200	H	-5.48268200	-2.13826200	0.55522900
H	-5.08557800	-0.69306400	-1.43671100	H	-5.90335700	-0.76834800	-0.43969600
H	1.44207200	0.16397500	1.94581600	H	-0.83030500	1.63585200	0.43566800
H	5.52824100	-0.51204400	0.00449500	H	3.46552100	-4.03514700	0.06849600
C	0.31313100	1.21353200	0.45571100	C	1.16713100	2.10326300	-0.19811600
C	-0.96026500	1.50888900	-0.03940000	C	1.75596600	2.53035500	-1.38827200
C	1.39394700	1.99879200	0.04652700	C	1.72746400	2.50559600	1.01790200
C	-1.14664200	2.56034200	-0.93420900	C	2.88198600	3.35372800	-1.36784600
H	-1.81076700	0.91156900	0.27547600	H	1.32736900	2.22554300	-2.33808000
C	1.21139600	3.05456000	-0.84473500	C	2.85113200	3.32629100	1.04019300
H	2.38630500	1.78422200	0.43191500	H	1.26776700	2.17859100	1.94610600
C	-0.05992700	3.33494900	-1.34204000	C	3.43070100	3.75392800	-0.15392300
H	-2.13932600	2.77553600	-1.31272300	H	3.32542700	3.68243000	-2.30051300
H	2.05958200	3.65622200	-1.14951400	H	3.27245100	3.63783200	1.98906200
H	-0.20491900	4.15370100	-2.03649500	H	4.30291300	4.39647500	-0.13628800
H	-3.35243200	-3.20370700	0.91021800	H	2.15120200	-2.65633200	-2.24029400
H	3.83559400	-1.79479300	-2.08206500	H	-3.55321300	-1.38570200	2.35716200
H	4.55166300	0.01001000	1.89514500	H	3.19939900	-3.27837100	2.00429300
O	5.32901500	0.39179800	1.46900000	O	3.90985000	-3.86577500	1.71433000
H	5.29392000	1.34091300	1.62145800	H	4.73213400	-3.48992700	2.04327300
O	-0.32993800	4.18032900	2.07028800	H	0.59161400	4.82634500	-0.85761800
H	-0.80569500	3.84838400	1.30076000	O	0.24234100	5.35085600	-0.12899700
H	0.52361300	3.73922100	2.01598400	H	0.63433500	4.94066700	0.64909800
H	-5.23944600	2.09896400	-1.05594700	H	-5.11245800	1.10131000	-1.11814300
O	-5.48160400	1.23223200	-0.71935200	O	-6.03115100	1.09981300	-1.43770400
H	-4.77358900	0.97323500	-0.10742200	H	-5.98757100	1.31618600	-2.37264400
H	-0.04920100	-0.20161400	-2.49511600	H	-1.17558500	1.90900100	3.30687600
O	0.74173500	0.24578400	-2.83451900	O	-0.68925300	1.09173800	3.15694200
H	0.62832800	1.17472900	-2.60141000	H	-0.33440900	0.84802700	4.01809200
H	-2.90834100	-0.60494000	2.28400900	H	-0.47566200	-1.64784600	-2.34515900
O	-2.37147400	-0.91051000	3.03591100	O	0.18173400	-1.53835000	-3.05587100
H	-2.90843500	-0.78102300	3.82183600	H	-0.31086900	-1.49583300	-3.87940700

**Inverse γ-turn with $g^+g^\pm$ side chain ΔE=+1.78**			
C	-0.01120000	3.84790000	0.06700000
C	-0.24520000	2.76430000	-0.97460000
O	-1.10360000	2.94410000	-1.83630000
H	0.55110000	4.66210000	-0.38880000
N	0.51450000	1.67340000	-0.86690000
C	0.45750000	0.57370000	-1.82190000
C	-0.70400000	-0.39210000	-1.51640000
O	-0.54420000	-1.60690000	-1.44940000
C	1.81390000	-0.14400000	-1.90280000
H	1.02530000	1.53550000	0.00950000
H	0.23130000	1.01000000	-2.79970000
H	1.66130000	-1.08140000	-2.43850000
N	-1.91060000	0.18050000	-1.36040000
C	-3.06860000	-0.64080000	-1.08770000

O	-2.68780000	-0.52200000	1.28070000
H	-2.02030000	1.17020000	-1.54860000
H	-3.95640000	-0.00890000	-1.12910000
N	-1.35480000	4.35170000	0.45530000
H	-1.82310000	3.60570000	1.00250000
H	-1.30260000	5.20460000	1.01350000
C	-3.00630000	-1.23170000	0.31660000
N	-3.36400000	-2.50630000	0.43860000
H	-3.40100000	-3.10260000	-0.38230000
H	-3.34070000	-2.92560000	1.36350000
H	2.47850000	0.48040000	-2.50190000
H	-1.90780000	4.53830000	-0.38730000
C	2.46240000	-0.40230000	-0.56050000
C	3.47820000	0.44590000	-0.10960000
C	2.06440000	-1.46380000	0.25940000
C	4.08250000	0.24640000	1.13030000
H	3.80080000	1.27070000	-0.73700000
C	2.66570000	-1.66490000	1.50030000
H	1.28450000	-2.13370000	-0.08150000
C	3.67450000	-0.80960000	1.94170000
H	4.86860000	0.91490000	1.46090000
H	2.35020000	-2.49540000	2.12120000
H	0.50120000	3.48620000	0.95520000
H	-3.17110000	-1.43180000	-1.83190000
H	4.14320000	-0.97010000	2.90540000
H	-3.22520000	-2.28120000	4.13180000
O	-2.74180000	-2.42730000	3.31460000
H	-2.67970000	-1.56130000	2.87960000
O	-1.69510000	1.93120000	1.80350000
H	-2.08640000	2.03750000	2.67870000
H	-2.08460000	1.09740000	1.45650000
H	-1.58970000	-3.11740000	-1.83980000
O	-2.27600000	-3.80380000	-1.88600000
H	-2.35290000	-4.04230000	-2.81350000
H	4.31960000	-2.89190000	-0.49400000
O	5.16770000	-2.51250000	-0.74480000
H	5.10230000	-1.59790000	-0.44800000
H	0.15180000	1.42750000	2.11340000
O	1.10540000	1.48150000	1.93790000
H	1.49620000	0.65760000	2.25020000

*Calculations at the DFT/M062X/6-311++G(d,p) level

Table S5 Atomic cartesian coordinates of the 3 lowest energy conformers of cationic Gly-Tyr-Gly+5 water molecules*

Extended chain with tg± side chain	ΔE=0			Helical with g+g+ side chain	ΔE=+0.39		
C	-5.43421200	0.21750700	-0.31088300	N	-2.89344600	-3.41756600	-1.75858200
C	-4.03031400	-0.19783800	0.11568400	H	-3.20270600	-2.53210700	-2.16504200
O	-3.85774700	-1.11539600	0.91100900	C	-1.55510700	-3.28057600	-1.13619800
H	-5.49370900	0.37480700	-1.38678100	H	-3.59373600	-3.68855600	-1.02813200
N	-3.05759700	0.53656600	-0.42831500	H	-2.88416100	-4.12180400	-2.49761300
C	-1.65826600	0.27215000	-0.13924500	C	-1.63689100	-2.21898800	-0.05175100
C	-0.87797700	1.56320600	-0.37202300	H	-1.27126100	-4.24421000	-0.71437500
O	-1.08924700	2.24651700	-1.37334500	O	-2.69020300	-1.63282800	0.18029200
C	-1.11245500	-0.84087700	-1.06484700	N	-0.49151100	-1.97064100	0.59378300
H	-3.29045500	1.19526900	-1.17382500	H	0.35740600	-2.41816700	0.27100100
H	-1.57659000	-0.04798200	0.90165700	C	-0.40199900	-0.95505800	1.62872700
H	-1.80915100	-1.67924400	-0.99870200	H	-1.12753100	-1.17903400	2.41371500
N	0.05142500	1.85122200	0.54629400	C	-0.77156200	0.44028100	1.12394000
C	0.96966100	2.95711900	0.38297900	O	-1.15257300	1.28810700	1.92642400
O	2.71721000	1.62810500	1.36141700	N	-0.64730100	0.67812800	-0.19112400
H	0.19180900	1.20369500	1.32156100	H	-0.19200000	-0.00447700	-0.79725800
H	0.80164700	3.40105200	-0.59691900	C	-0.96339800	1.98394700	-0.71792200
N	-6.39402600	-0.84204700	0.08721400	C	-2.45204300	2.31396300	-0.69947700
H	-6.36115500	-0.98855700	1.12590100	H	-0.45161400	2.77296500	-0.16170700
H	-7.34670300	-0.58978000	-0.18017000	O	-2.79825800	3.49620000	-0.80920300
C	2.41291600	2.50496800	0.54599000	N	-3.31060300	1.30138800	-0.58606700
N	3.31735100	3.13993200	-0.20062300	H	-2.99706300	0.35100300	-0.42779200
H	3.05075700	3.85036000	-0.86389100	H	-4.30304000	1.51366000	-0.56857400
H	4.30104600	2.92695900	-0.07559400	C	1.01186800	-0.94516600	2.24391300
H	-1.13388700	-0.46539800	-2.09198200	H	1.01838200	-0.17723700	3.01985500
H	-6.16849600	-1.73286600	-0.36004600	H	1.17137900	-1.90918400	2.73130100
C	0.27219700	-1.28821800	-0.67755000	C	2.09758600	-0.68703800	1.22724000
C	0.45626800	-2.40924300	0.13599300	C	2.38086400	0.61158500	0.79068400
C	1.40530600	-0.57723700	-1.07833200	C	2.82563600	-1.73800200	0.66434100
C	1.72634200	-2.80901900	0.54078100	C	3.34249600	0.85899100	-0.18097700
H	-0.40761200	-2.98382100	0.45642800	H	1.84721600	1.45141200	1.22632100
C	2.68255100	-0.95975400	-0.68078000	C	3.79162800	-1.51049500	-0.31427600
H	1.29392300	0.29169600	-1.72182900	H	2.65316800	-2.75571800	1.00303600
C	2.85085100	-2.08261400	0.13596200	C	4.05408500	-0.20776100	-0.74498900
H	1.86395200	-3.68492400	1.16416800	H	3.55504300	1.87132000	-0.50588100
H	3.55109100	-0.39535800	-1.00224600	H	4.35584900	-2.33059000	-0.74218200
H	-5.71640400	1.13803900	0.19999500	H	-0.82530800	-2.98327000	-1.89012500
H	0.79007800	3.72568100	1.14163200	H	-0.63069000	2.03365600	-1.75568000
O	-5.92048200	-1.01310000	2.77608900	H	-4.33328200	-3.02211000	0.90198000
H	-6.31534700	-1.56536700	3.45750700	O	-4.34771600	-3.89792800	0.49750800
H	-5.00340300	-1.29999900	2.67256200	H	-5.21845400	-4.26994800	0.66862100
O	1.77991600	-3.51051700	-2.84336700	H	4.97531000	-0.96594600	2.06500000
H	1.39467200	-3.76358000	-1.99773000	O	5.27680800	-0.16732800	2.51085200
H	2.05840300	-2.59979700	-2.69740300	H	4.72386800	0.52533200	2.13403800
H	4.52142400	1.37321100	1.40969900	O	-5.55483400	3.20514300	-0.60012900
O	5.43783700	1.45112300	1.08130000	H	-6.13770300	3.54663200	-1.28319000
H	5.98301700	1.68288400	1.83946700	H	-4.68408500	3.60577100	-0.76404900
H	0.93639800	-1.02628100	2.15669900	H	-1.62454400	3.13235100	1.97662100
O	0.91801100	-0.13968500	2.53459100	O	-1.86755000	4.07064100	1.94927200
H	1.72915900	0.28932100	2.21604800	H	-2.22114200	4.19595800	1.06004100
O	-3.51930200	2.26557700	-2.72652200	H	0.86161200	-0.92525800	-2.89616000
H	-4.00459500	3.09239600	-2.79619400	O	0.83466000	-1.13650100	-1.95686500
H	-2.60378500	2.50812100	-2.51587100	H	1.76018900	-1.17246400	-1.67966200
O	4.06213800	-2.50959900	0.54711300	O	5.00130400	-0.02990500	-1.68739500
H	4.77381000	-1.92303800	0.20438800	H	5.09563000	0.91924300	-1.90835400
O	5.94843800	-0.81953000	-0.36818700	H	4.95545200	2.95494600	-3.11793900
H	5.90679800	0.01015700	0.14773000	O	5.22107800	2.63082400	-2.25145800
H	6.87184900	-1.08624300	-0.39139600	H	6.04713100	3.08117400	-2.04780300

Inverse γ-turn with g⁻g⁺ side chain ΔE=+0.65			
C	0.20737700	-2.88427300	0.48120600
C	0.57465400	-1.57148000	-0.19234300
O	1.31067000	-1.57103800	-1.17595300
H	-0.78826000	-3.19797000	0.16899500
N	0.03587000	-0.47488400	0.34842300
C	0.10561100	0.81964700	-0.32336800
C	1.39197600	1.56244800	0.07533200
O	1.36692900	2.62735000	0.68573400
C	-1.12919300	1.65729900	-0.00160800
H	-0.63488000	-0.60301200	1.10687700
H	0.17288300	0.62386700	-1.39801500
H	-1.06299600	2.00015600	1.03340700
N	2.52999100	0.95729300	-0.29052300
C	3.81492000	1.51591800	0.05859500
O	4.65245700	-0.67936100	-0.43026400
H	2.49367200	0.07218300	-0.78995400
H	3.99757600	2.45530700	-0.47094300
N	1.20733400	-3.89077100	0.04935900
H	2.12824900	-3.59837800	0.43902600
H	0.96774200	-4.82995000	0.36715800
C	4.91233000	0.51751100	-0.25496200
N	6.14916500	1.00584400	-0.30923600
H	6.33175100	1.98993800	-0.18845300
H	6.92266500	0.36703800	-0.47100800
H	-1.09236200	2.55549600	-0.62448700
H	1.28812000	-3.89095600	-0.97065900
C	-2.43620700	0.92352400	-0.20288300
C	-3.49954900	1.15461600	0.67115200
C	-2.63221500	-0.00036600	-1.23442100
C	-4.72018500	0.49595700	0.53180700
H	-3.37358500	1.85730100	1.48868300
C	-3.84162300	-0.66732100	-1.38900000
H	-1.83204700	-0.21597000	-1.93513800
C	-4.89622400	-0.42471800	-0.50471300
H	-5.53440900	0.68767000	1.22152000
H	-3.98170100	-1.38650500	-2.18738700
H	0.23497900	-2.80500300	1.56536000
H	3.85458500	1.73830800	1.13061300
H	3.04594400	5.15479400	1.86937900
O	3.43656500	4.37276600	1.47156500
H	2.68455600	3.81343900	1.21550600
H	-4.54368500	2.65915700	-1.26286300
O	-4.58588400	2.62783900	-2.22447500
H	-4.24575600	1.75146700	-2.43533800
O	3.10158400	-2.33059900	1.18486900
H	3.62045200	-1.74026400	0.60814300
H	3.62480600	-2.46083400	1.98087300
O	7.24872800	-1.58449100	-0.98382700
H	6.29879700	-1.66222500	-0.80514300
H	7.66357500	-2.34295400	-0.56462400
H	-2.93845500	-1.09024700	1.62277500
O	-2.12886600	-1.52633500	1.91684700
H	-2.29176600	-1.78072400	2.83049500
O	-6.05043600	-1.10028000	-0.69252000
H	-6.71158700	-0.84741700	-0.01718300
H	-8.67873900	0.07501100	0.94507400
O	-7.86087200	-0.35313100	1.21737800
H	-8.11367200	-0.98541200	1.89756200

*Calculations at the DFT/M062X/6-311++G(d,p) leve

Table S5 Atomic cartesian coordinates of the 3 lowest energy conformers of cationic Gly-Tyr-Gly+5 water molecules*

**pP-II with g^+g^+ side chain**	**ΔE=0**			**Extended chain with tg^+ side chain**	**ΔE=+1.12**		
N	-2.56481400	-4.34727300	-1.53433800	C	5.50759100	0.65624000	0.92067000
H	-3.36554100	-4.49975800	-0.91455000	C	4.11363600	0.09432200	1.16108200
C	-1.37394500	-3.97416300	-0.73253800	O	3.95376400	-1.09546800	1.38716400
H	-2.40825300	-5.18486000	-2.09495200	H	5.88593900	1.14518100	1.81744300
H	-2.78727900	-3.53370400	-2.15023400	H	5.50547400	1.35468200	0.08387900
C	-1.81647900	-2.87610300	0.22565500	N	3.11158700	0.98750800	1.10082900
H	-1.02776900	-4.84279000	-0.17345900	C	1.73888400	0.51677200	1.04655100
H	-0.59139500	-3.62484600	-1.40538100	C	1.58575700	-0.45917200	-0.12609100
O	-2.99523300	-2.75810700	0.52493300	O	2.19479200	-0.26482900	-1.18879500
N	-0.83414300	-2.09879900	0.70966800	H	3.30904800	1.87794700	0.65441400
H	0.05461600	-2.10637500	0.22000700	H	1.48760900	0.00500800	1.97873700
C	-1.16641900	-0.92418300	1.49160200	N	0.73641000	-1.46304100	0.05931000
H	-1.77850400	-1.22309000	2.34424400	C	0.35095200	-2.35330100	-1.01134600
C	-1.97938000	0.05542000	0.63434000	O	-1.81764800	-2.41080300	0.03705200
O	-1.91484700	0.05900600	-0.59902600	H	0.27838900	-1.55634500	0.96648700
N	-2.73533600	0.92453200	1.30792300	H	0.60015100	-1.88403300	-1.96412200
H	-2.74516300	0.89707100	2.32671200	H	0.89526800	-3.30163100	-0.95166500
C	-3.49719200	1.91761300	0.58996800	N	6.37936500	-0.49358000	0.57737000
C	-2.59112500	2.89909300	-0.14773800	H	6.04065300	-0.87957600	-0.32913700
H	-4.17750100	1.43580600	-0.11502700	H	7.35977500	-0.22240200	0.50066900
H	-4.09054300	2.48759300	1.30608700	C	-1.13765600	-2.66700400	-0.95523100
O	-1.49776200	3.22257600	0.31446900	N	-1.62703900	-3.29072800	-2.03010000
N	-3.08251600	3.40300800	-1.28529300	H	-1.07165300	-3.44007800	-2.85817800
H	-3.96098100	3.09043200	-1.66752900	H	-2.60681700	-3.53284900	-2.05869100
H	-2.53570600	4.07621200	-1.80755900	H	6.28913900	-1.22924500	1.28299400
C	0.11878000	-0.25142200	2.01619500	C	0.81624000	1.73882100	0.85590100
H	-0.17725900	0.63884500	2.57444600	H	1.01919200	2.42481200	1.68177400
H	0.58429000	-0.93723800	2.72853800	H	1.11005500	2.24668800	-0.07006400
C	1.08450200	0.10369200	0.92462600	C	-0.64001400	1.39317600	0.81984500
C	2.15892100	-0.70754800	0.41423200	C	-1.38553000	0.89679500	-0.30713300
C	1.05732300	1.23469500	0.14299200	C	-1.52383100	1.43691200	1.87326500
C	2.73065000	0.00140000	-0.67397900	C	-2.70350600	0.65528900	0.14494700
C	2.67144100	-1.97202600	0.75352600	C	-1.05747200	0.62073800	-1.64602100
H	0.38163300	2.07756500	0.19755800	H	-1.35620800	1.75935200	2.89134300
C	3.78471300	-0.52347300	-1.43136300	C	-3.69957300	0.14494300	-0.69716400
C	3.70897700	-2.49575200	-0.00000600	C	-2.03435600	0.10129500	-2.47618200
H	2.25846600	-2.53298400	1.58572500	H	-0.05424900	0.79846400	-2.02066000
H	2.19953500	1.91872300	-1.49750100	H	-3.58293100	0.85394300	2.05545600
C	4.25832900	-1.77693800	-1.08404700	C	-3.34463400	-0.13533900	-2.00387900
H	4.20719100	0.02921100	-2.26220400	H	-4.70435100	-0.03026000	-0.33249900
H	4.10843300	-3.47325700	0.24244500	H	-1.79564300	-0.12890800	-3.50820700
H	5.06896400	-2.21642700	-1.65291000	H	-4.08592400	-0.54042400	-2.68283400
N	2.03981200	1.17882600	-0.80988600	N	-2.75935500	0.99839200	1.47250500
H	-0.60159100	4.31280400	-0.78081400	H	-3.59792400	-2.38803700	0.51011100
O	-0.29623200	4.76284800	-1.59382700	O	-4.45087500	-2.42782300	0.97851800
H	-0.18093600	5.68906300	-1.36124300	H	-4.46318400	-3.28817600	1.40844100
H	-3.16618600	0.12090200	4.68028800	H	3.07801000	1.38794600	-1.55020200
O	-2.45535500	0.49557500	4.15061100	O	3.62173600	2.18221200	-1.41756800
H	-2.00039000	1.11090800	4.73414700	H	3.50658200	2.72290300	-2.20343800
O	0.08285200	-1.26292500	-2.07312500	H	-1.48535700	-1.85662100	1.81911700
H	-0.35715000	-0.60315900	-1.50842300	O	-0.86281500	-1.69584400	2.54562000
H	0.93032000	-0.88388200	-2.32810700	H	-1.13923700	-0.86389600	2.94147100
H	-1.62346900	-1.70213200	-2.86332800	H	3.92177800	-0.87525100	-1.52784000
O	-2.56176800	-1.85456500	-2.66362700	O	4.87334400	-1.03446100	-1.65158200
H	-2.74143100	-1.19979900	-1.97169700	H	5.08965200	-0.67927400	-2.51913600
H	1.18809100	3.95994500	-2.25022400	H	-6.02854700	0.02389400	2.36855400
O	1.96142400	3.44282900	-2.54696200	O	-5.10440200	-0.20442000	2.49847600
H	2.68033100	4.07474900	-2.63414400	H	-4.95047500	-1.02159800	1.98660400
H	2.69585400	1.52836000	1.96905700	H	-1.73428600	3.44649200	0.60357800

O	3.52389600	1.82985400	2.35976300	O	-2.29780400	4.12551300	0.20759700
H	4.18286900	1.17965300	2.06454800	H	-1.79503100	4.94317500	0.27116400
H	5.81600200	0.18029400	0.65220800	O	-3.27527500	3.29910900	-2.30822100
O	5.44417100	-0.06650400	1.50541600	H	-2.92814800	3.57273800	-1.44320100
H	4.97568500	-0.89242200	1.32698000	H	-3.08099000	2.35709000	-2.36314400

*Calculations at the DFT/M062X/6-311++G(d,p) level
